# Multicolor Luminescent Supramolecular Bidirectional Shuttles Driven by Light

**DOI:** 10.1002/advs.202507090

**Published:** 2025-06-04

**Authors:** Rong Zhang, Zhuo Lei, Zhiyi Yu, Yong Chen, Yu Liu

**Affiliations:** ^1^ College of Chemistry State Key Laboratory of Elemento‐Organic Chemistry Nankai University Tianjin 300071 P. R. China

**Keywords:** cucurbituril, multicolor luminescent, photo‐responsive, supramolecular assembly

## Abstract

Possessing special photophysical behavior, supramolecular space‐confined assembly is widely applied in bioimaging, information anti‐counterfeiting, and luminescent materials. Herein, two different multicolor luminescent supramolecular bidirectional shuttles driven by light are reported, which are constructed by spiropyran‐modified *p*‐styrene derivatives (BPSP), cucurbit[7]uril (CB[7]) and cucurbit[8]uril (CB[8]). CB[7] encapsulated BPSP not only effectively enhance the fluorescence intensity both in the ring‐closed and ring‐open states accompanied with multicolor luminescence from blue to red, but also produce ortho‐hexagonal nanosheets. Different from CB[7], the binding of CB[8] to BPSP formed a nanospherical topological morphology. It induce the red‐shift of fluorescence from blue to yellow, which completely transform into red fluorescence in the dark. Both the multicolor luminescence and topological morphology can be reversibly regulate under alternating visible light and darkness. Due to the photo‐controlled time‐dependent multicolor reversible luminescence of the two kinds of supramolecular assemblies, they are successfully applied to logic gates, bidirectional reversible anti‐counterfeiting, and controllable cell imaging, providing a new strategy for macrocycle‐confined molecular assembly.

## Graphical abstract

Two different multicolor luminescent supramolecular bidirectional shuttles driven by light is constructed by spiropyran‐modified *p*‐styrene derivatives (BPSP) and cucurbit[7]uril and cucurbit[8]uril, which not only realize photo‐controlled time‐dependent multicolor luminescence from blue to red, but also achieve the regulation of topological morphology under alternating visible light irradiation and darkness. They are successfully applied to logic gates, dynamic information encryption, and tunable cell imaging.

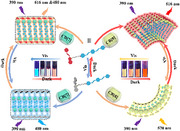

## 1 Introduction

Intelligent luminescent systems have attracted extensive attention due to their wide range of applications in light‐emitting diodes,^[^ ^1^ ^]^ information anti‐counterfeiting,^[^ ^2^, ^3^, ^4^, ^5^ ^]^ bioimaging,^[^ ^6^ ^]^ sensing and detection.^[^ ^7^, ^8^, ^9^, ^10^, ^11^, ^12^ ^]^ Among the various approaches to construct luminescent systems, supramolecular assembly strategies undoubtedly have significant advantages due to their simplicity and dynamic controllability, which provide flexibility and diversity for the design and functionalization of luminescent systems.^[^ ^13^, ^14^, ^15^, ^16^ ^]^ In particular, supramolecular space‐confined assembly based on dynamically adjustable non‐covalent forces including host‐guest interaction, hydrogen bond interaction, and coordination interaction can not only confer photophysical properties not originally possessed by the precursor compound,^[^ ^17^, ^18^ ^]^ such as inducing the luminescence of guest molecules, but also expand the luminescence range through cascade energy transfer, thus enabling realize multicolor luminescence behavior.^[^ ^19^, ^20^, ^21^, ^22^, ^23^, ^24^, ^25^ ^]^ For example, Yang reported a novel macrocycle fluoren‐triangle constructed by fluoren and resorcinarene backbone.^[^ ^26^ ^]^ The macrocycle molecule had a triangular conformation in the solid state and exhibited aggregation‐induced luminescence and excitation‐dependent luminescence properties, which produced multicolor fluorescece from dark blue, green to orange. In addition, the fluoren‐triangle could selectively adsorb and separate gases. Qu group described a time‐dependent multicolor fluorescence coordination assembly system. in which blue fluorescent 2,6‐pyridinedicarboxylic acid‐substituted pyrene derivative Py(DPA)_2_ in aqueous solution was coordinated with zinc ions and assembled into nanofibers with yellow fluorescence.^[^ ^27^ ^]^ This metastable structure gradually transformed into thermodynamically stable nanosheets over time and emitted green fluorescence. Taking advantage of the time‐dependent multicolor luminescence characteristics, the assembly system was applied to information encryption materials. Our group reported a tunable hierarchical supramolecular assembly system, which was composed of tetraphenylethylene pyridinium (TPE‐Py), sulfobutylether‐β‐cyclodextrin (SBE‐βCD) and cucurbit[8]uril (CB[8]). The primary assembly TPE‐Py@SBE‐βCD and the secondary assembly TPE‐Py⊂CB[8]@SBE‐βCD emitted different colors of fluorescence, and further introduction of the near‐infrared dye AlPcS4 into TPE‐Py⊂CB[8]@SBE‐βCD produced even more red‐shifted fluorescence emission.^[^ ^28^ ^]^ This multicolor luminescence regulated by multi‐level assembly has been successfully applied in logic gates and information storage.

**Scheme 1:**
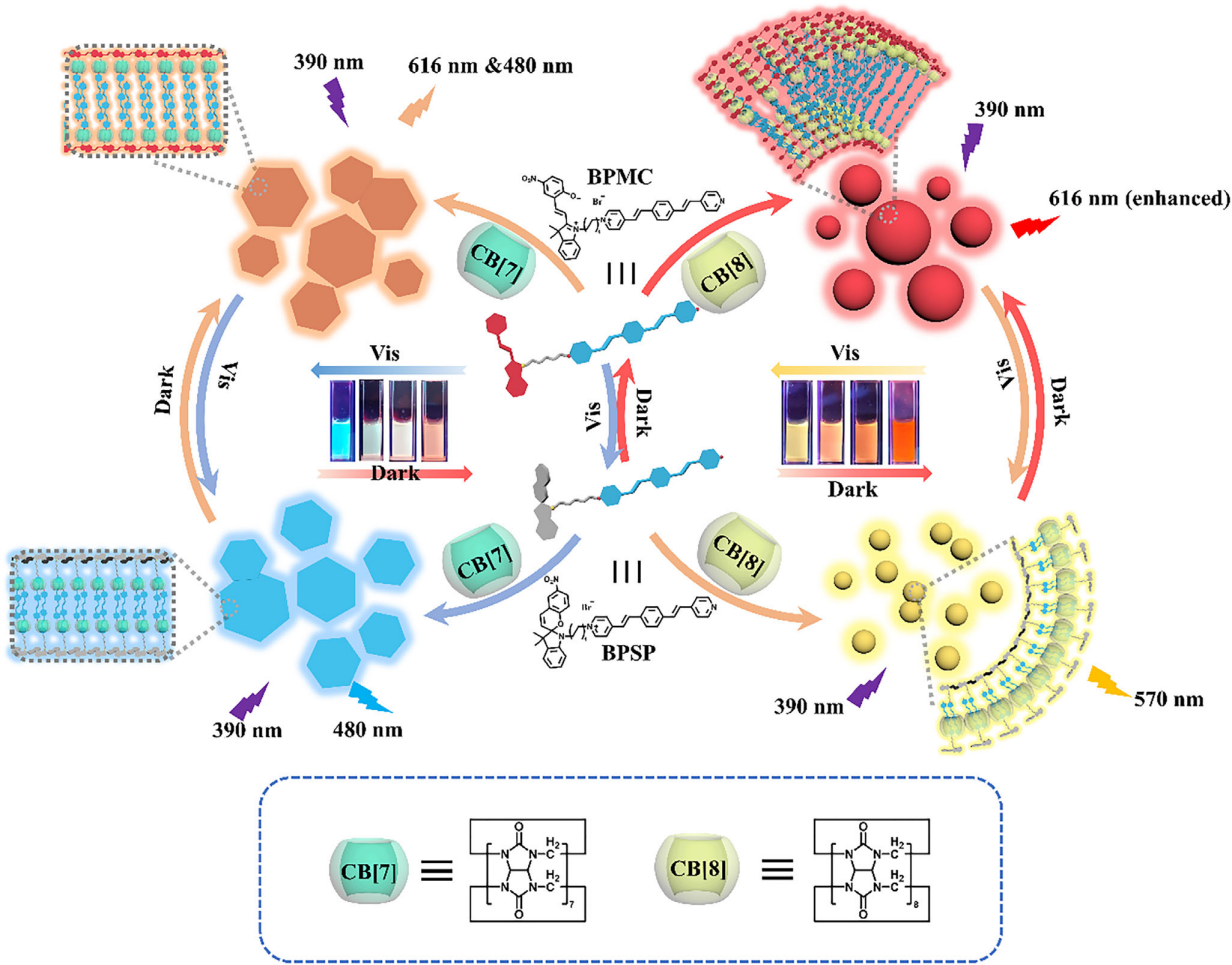
Schematic illustration for constructing photo‐controlled time‐dependent multicolor luminescent supramolecular shuttle with tunable topological morphology.

Although the supramolecular assembly‐induced multicolor luminescence system has been greatly developed, it is still a research hotspot to construct an intelligent response supramolecular system to external stimuli such as light, heat, electricity, magnetism, mechanical force, pH, and biological enzymes and achieve reversible regulation of the assembly‐induced multicolor fluorescence.^[^ ^29^, ^30^, ^31^, ^32^, ^33^, ^34^, ^35^ ^]^ The integration of non‐invasive, space‐time controllable light responsiveness and macrocycle confined regulation of multicolor fluorescence to obtain more complex and variable luminescence behavior of supramolecular systems has rarely been reported. Herein, we constructed cucurbituril‐modulated and photo‐regulated reversible multicolor fluorescent supramolecular assemblies with distinct topological morphologies, which were assembled from spiropyran‐modified *p*‐styrene derivatives (BPSP), cucurbit[7]uril (CB[7]) and cucurbit[8]uril (CB[8]) through host‐guest interactions (**Scheme** **1**). Due to the photoisomerization of the spiropyran moiety, the CB[7]‐encapsulated ring‐closed state BPSP under visible light irradiation effectively enhanced the blue fluorescence at 480 nm and formed the topological morphology of the regular hexagonal nanosheets. Under dark conditions, ring‐closed BPSP was converted into ring‐open form BPMC, and CB[7] simultaneously enhanced the fluorescence emission both at 480 and 616 nm. For CB[8] with a larger cavity size, it could induce the bathochromic shift of ring‐closed BPSP from blue fluorescence at 480 nm to yellow fluorescence at 570 nm and had a small nanoparticle morphology, which was completely converted into red fluorescence at 616 nm and produced a nanosphere in the dark environment due to the change of binding mode. It not only realized the regulation of topological morphology, but also achieved two photo‐controlled time‐dependent multicolor luminescent supramolecular shuttles under alternating visible light irradiation and darkness, which has been successfully applied in logic gates, dynamic information encryption, and tunable bioimaging.

## 2 Results and Discussion

### 2.1 The Assembly Behavior of the Two Supramolecular Systems with Different Topological Morphologies

**Figure 1:**
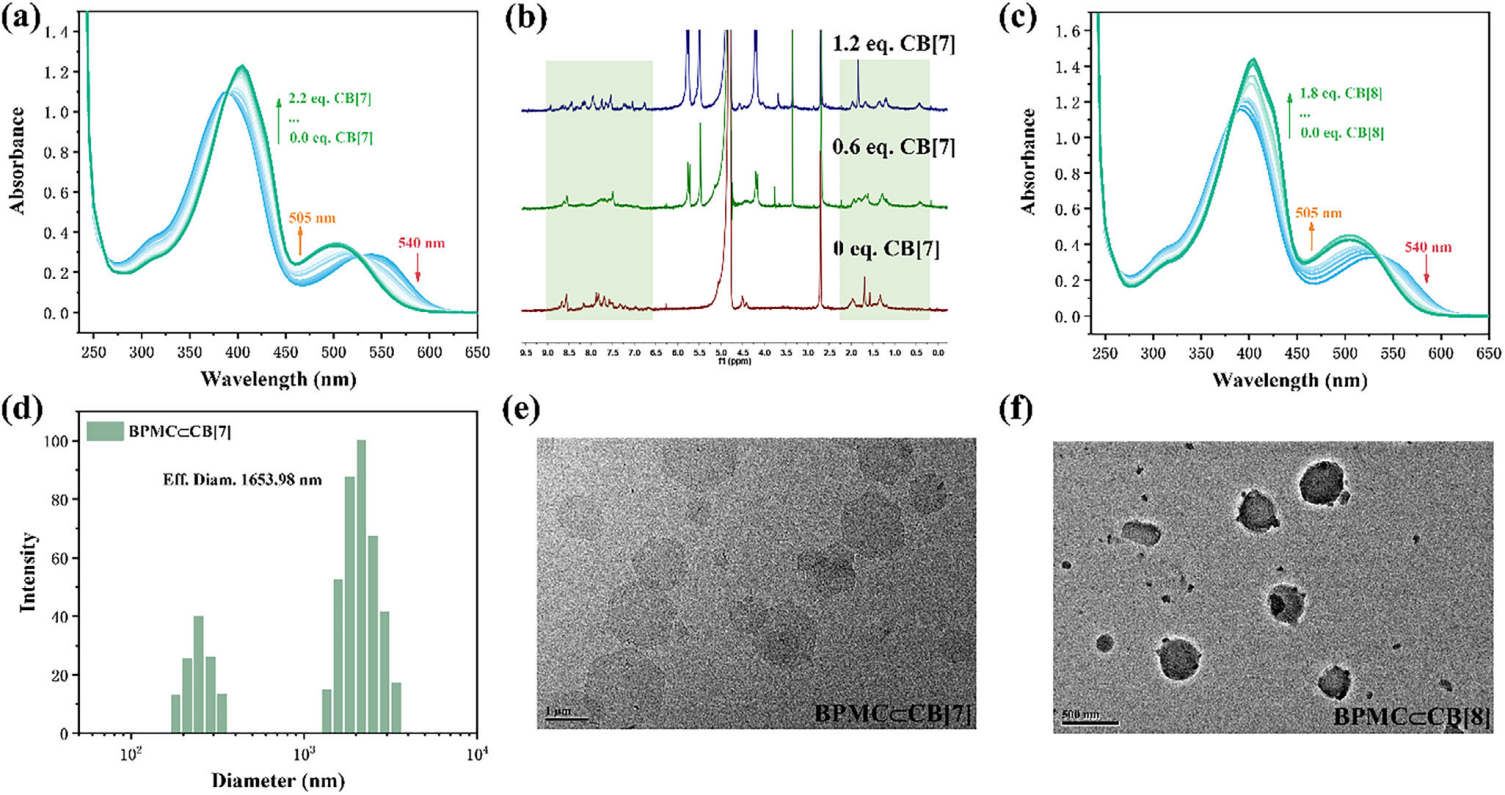
a) UV‐vis absorption spectra of BPMC with the addition of CB[7] ([BPMC] = 2.0 × 10^−5^ M, [CB[7]] = 0–4.4×10^−5^ M). b) ^1^H NMR spectra of BPMC with the addition of CB[7] (D_2_O:DMSO‐*d_6_* = 20:1, [BPMC] = 5.0×10^−4^ M, [CB[7]] = 0, 3 × 10^−4^, 6 × 10^−3 ^M). c) UV‐vis absorption spectra of BPMC with the addition of CB[8] ([BPMC] = 2.0 × 10^−5^ M, [CB[8]] = 0–3.6 × 10^−5^ M). d) DLS of BPMC⊂CB[7] ([BPMC] = 2.0 × 10^−5^ M, [CB[7]] = 2.0 × 10^−5^ M). TEM images of e) BPMC⊂CB[7] and f) BPMC⊂CB[8] ([BPMC] = [CB[7]] = [CB[8]] = 2.0×10^−5^ M).

The guest molecule BPSP was synthesized by substitution reaction according to the synthetic route of Scheme S1 (Supporting Information) and its structure was characterized by nuclear magnetic resonance spectroscopy (^1^H NMR, ^13^C NMR) and high‐resolution mass spectrometry (HRMS) (Figures S1–S3, Supporting Information). Due to the high affinity of the structurally rigid cucurbituril to organic cations, the host‐guest behavior of two cucurbiturils CB[7,8] and the cationic guest BPSP was first investigated by UV‐vis absorption spectra. As shown in Figure S4a (Supporting Information), a characteristic absorption peak centered at 394 nm of BPSP was observed in the UV‐vis absorption spectrum after pretreatment with visible light irradiation at 530 nm for 30 s, which declined and bathochromic shifted to 405 nm as the concentration of CB[7] in aqueous solution increased gradually. When the concentration of CB[7] reached 1 equivalent of BPSP, the increase in the intensity of 405 nm absorption peak slowed down and gradually reached equilibrium. The binding constant (*K_a_*) of CB[7] to BPSP is 1.56 × 10^6^ M^−1^ according to the change of absorption value at 405 nm by nonlinear curve fitting (Figure S4b, Supporting Information). In addition, the Job's plot showed that the binding ratio of CB[7] to BPSP was 1:1 (Figure S5, Supporting Information). In the dark environment, the guest molecule isomerized from the ring‐closed BPSP form to the ring‐open BPMC form, and from the Job's results, it was clear that the binding ratio of CB[7] to BPMC was still 1:1 (Figure S6, Supporting Information). According to the UV‐vis absorption spectra (**Figure** **1**a), it was observed that not only the bathochromic shift and enhanced intensity of characteristic absorption peak at 394 nm, but also the absorption peak at 540 nm belonging to the ring‐open BPMC exhibited a hypsochromic shift to 505 nm during the gradual addition of CB[7] to the BPMC aqueous solution. A nonlinear curve fitting of the change in absorption value at 505 nm yielded a complex constant (*K_a_*) of 3.97×10^7^ M^−1^ for CB[7]⊂BPMC (Figure S7, Supporting Information), indicating that CB[7] has a similar binding behavior to both the ring‐closed BPSP and the ring‐open BPMC. Furthermore, ^1^H NMR titration experiments showed the upfield shift of alkyl chain protons in ring‐open BPMC with the addition of CB[7], while those on the styrylpyridine and the spiropyran moiety exhibited a downfield shift, demonstrating CB[7] predominantly encapsulated in the alkyl chain portion of BPMC (Figure 1b). Under visible light irradiation, the protons on the styrylpyridine showded the upfield shift, indicating that CB[7] tended to encapsulate the styrylpyridine moiety of BPSP (Figure S8, Supporting Information). The mass spectrometry (MALDI‐TOF) of the assembly BPMC⊂CB[7] showed the test result [BPMC+CB[7]‐Br]^+^ (1837.69) matched the calculated value (1837.67), proving the bonding ratio of BPMC and CB[7] (Figure S9, Supporting Information). Next, the host‐guest behavior of CB[8] encapsulated guest molecules was investigated. It was found that the bonding ratio of the ring‐closed isomer BPSP to CB[8] was 2:1 after pretreatment with visible light irradiation (Figure S10, Supporting Information). In the UV‐vis spectra (Figure S11, Supporting Information), the characteristic absorption peaks at 394 nm were observed to gradually decrease and red‐shift with the incorporation of CB[8], and based on the nonlinear function of the UV‐titration change at 394 nm, the complex constants of CB[8] for BPSP were calculated to be *K_a1_* = 1.54 × 10^3^ M^−1^ and *K_a2_* = 6.65 × 10^6^ M^−1^. As for the ring‐open form BPMC under dark conditions, the Job's plot showed the inflection point occurred at a transverse coordinate of 0.5, i.e., the bonding ratio of CB[8] to BPMC was 1:1 (Figure S12, Supporting Information). As shown in Figure 1c, the absorption peak at 394 nm of BPMC was gradually red‐shifted and increased, a blue‐shift of the absorption peak at 540 nm was also observed at the same time upon the concentration of CB[8] increasing. Finally, when the concentration of CB[8] was increased to one equivalent amount of BPMC, the absorption peak stabilized at 505 nm, and a fitting curve based on the variation of the absorption peak at 505 nm gave the bonding constant of CB[8] to BPMC, *K_a_* = 3.96×10^7^ M^−1^(Figure S13, Supporting Information). Further ^1^H NMR titration experiment was employed to characterize the encapsulation of CB[8] with two isomeric guest molecules, which revealed the protons on the *p*‐styrylpyridine of BPSP were shifted to the upfield, suggesting CB[8] was encapsulated of the *p*‐styrylpyridine portion of BPSP upon visible light irradiation (Figure S14, Supporting Information). In the dark, the proton signal was more significantly shifted toward the upfield, demonstrating that CB[8] was inclined to encapsulate the alkyl chain moiety of BPMC (Figure S15, Supporting Information). In the MALDI‐TOF mass spectra of BPMC⊂CB, [8] the experimental result [BPMC+CB[8]‐Br]^+^ (2003.63) was found to match the calculated value (2003.57), confirming the 1:1 bonding mode of BPMC with CB[8] (Figure S16, Supporting Information). While for BPSP⊂CB [8], the signal at 1340.09 should be attributed to [2BPSP+CB[8]‐2Br]^2+^ (Figure S17, Supporting Information), suggesting the 1:2 bonding mode of CB[8]⊂BPSP. Subsequently, the particle size and topological morphology of the assemblies were explored. Dynamic light scattering (DLS) results showed a micrometer‐scale hydration diameter (1.65 µm) of the BPMC⊂CB[7] assembly constructed in the dark (Figure 1d), which was significantly larger than the nanoscale particle size of BPMC itself (149.23 nm) (Figure S18, Supporting Information). The topological morphology of BPMC⊂CB[7] could be observed under transmission electron microscopy (TEM) and scanning electron microscopy (SEM) as a regular ortho‐hexagonal sheet with micrometer size (Figure 1e; Figure S19, Supporting Information), which was in accordance with the DLS results. The hydrated diameter of the BPSP⊂CB[7] assembly formed under visible light irradiation was reduced to 769.58 nm according to the DLS results (Figure S20a, Supporting Information), which was still significantly larger than the particle size of BPSP itself (136.76 nm), and the topological morphology of the BPSP⊂CB[7] in the TEM image was still the hexagonal nanosheet only with smaller size (Figure S20b, Supporting Information). These results indicated the encapsulation by CB[7] always generated assemblies with regular shapes and increased size regardless of whether the guest molecule was ring‐open or ring‐closed form. However, the incorporation of CB[8] produced a distinct topological morphology, as shown in Figure 1f, the BPMC⊂CB[8] constructed under dark conditions were nanosphere with a particle size of ≈300 nm in TEM as well as SEM images, which was in agreement with the DLS results (275.66 nm) (Figure S21, Supporting Information). The particle size of the BPSP⊂CB[8] decreased after visible light irradiation, and their morphological dissociation into small nanoparticles was observed in the TEM image, which should be related to the change in the assembly mode (Figure S22, Supporting Information).

### 2.2 Two Photo‐Controlled Time‐Dependent Multicolor Bidirectional Luminescence

**Figure 2:**
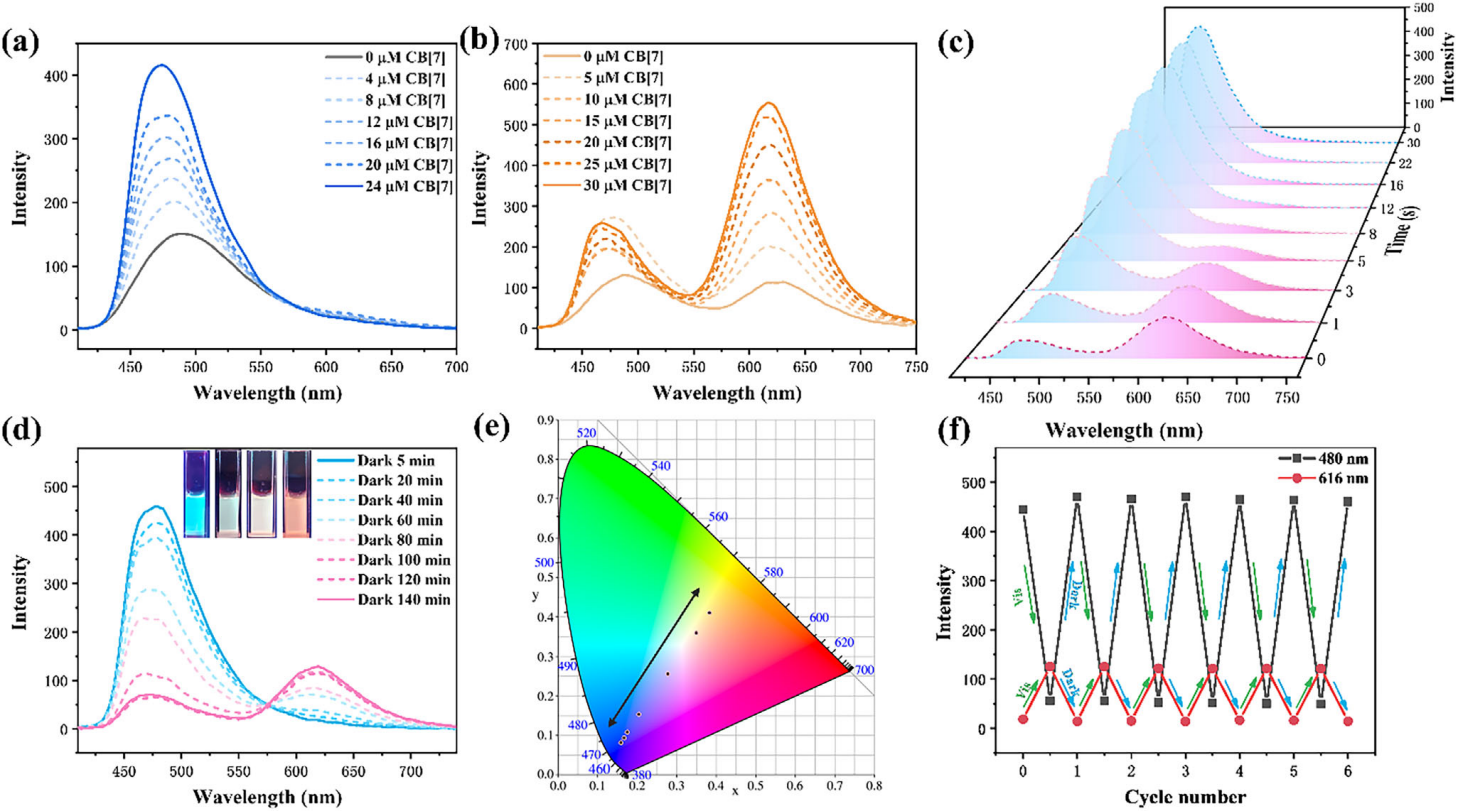
a) Fluorescence spectra of BPSP with the addition of CB[7] ([BPSP] = 2.0 × 10^−5^ M, [CB[7]] = 0–2.4 × 10^−5^ M). (b) Fluorescence spectra of BPMC with the addition of CB[7] ([BPMC] = 2.0×10^−5^ M, [CB[7]] = 0–3.0 × 10^−5^ M). c) Fluorescence spectra of BPMC⊂CB[7] upon visible light irradiation ([BPMC] = 2.0 × 10^−5^ M, [CB[7]] = 2.0 × 10^−5^ M). d) Time‐dependent fluorescence spectra of BPSP⊂CB[7] in the dark. Inset: fluorescence image of BPSP⊂CB[7] under UV portable light ([BPSP] = 2.0×10^−5^ M, [CB[7]] = 2.0×10^−5^ M). e) The CIE 1931 chromaticity diagram of BPSP⊂CB[7] in the dark. f) Fluorescence intensity at 480 nm and 616 nm of BPSP⊂CB[7] during cyclic darkness and visible irradiation ([BPSP] = 2.0×10^−5^ M, [CB[7]] = 2.0×10^−5^ M).

Since spiropyran moiety usually possessed reversible photoisomerization properties, the photo‐responsive properties of the guest molecules and corresponding assemblies with cucurbiturils were next investigated. An aqueous solution of the guest molecule was first placed in the dark for 2 h to obtain a light pink BPMC solution, and the UV absorption peaks of BPMC were explored for changes with visible light irradiation. According to the UV‐vis absorption spectra in Figure S23 (Supporting Information), the characteristic absorption peak at 540 nm gradually decreased under visible light irradiation and almost completely disappeared after 30 s of irradiation. In addition, a standard curve of the variation of absorption peak intensity at 540 nm with concentration was constructed based on the UV absorption spectra of BPMC at different concentrations (Figure S24, Supporting Information), and the reversible recovery efficiency of the guest molecules after the visible light irradiation‐darkness treatment cycle was calculated to be 97% (Figure S25, Supporting Information), indicating the excellent reversible conversion between the ring‐open form BPMC and the ring‐closed form BPSP could be achieved. In addition, the guest molecules exhibited photo‐modulated luminescence properties. BPSP emitted blue fluorescence centered at 480 nm at an excitation wavelength of 390 nm under visible light irradiation, whereas it was converted to ring‐open BPMC and emitted orange‐red fluorescence due to the presence of two fluorescence peaks at 480 and 616 nm (Figure S26, Supporting Information). The fluorescence intensities of BPSP and BPMC at different concentrations were further investigated, and the results showed a concentration of 2.0 × 10^−5^ M was both favorable for blue fluorescence of ring‐closed BPSP and red fluorescence emission of ring‐open BPMC (Figure S27, Supporting Information). Thus, the guest molecules at this concentration were selected for further assembly with cucurbituril to probe the luminescent behavior of the supramolecular assemblies. As shown in **Figure** **2**a, the gradual addition of CB[7] to the ring‐closed BPSP resulted in a significant enhancement of the initial fluorescence peak intensity by about 3‐fold accompanied by a slight blueshift. As for the ring‐open BPMC, the addition of CB[7] was able to not only enhance the intensity of fluorescence emission at 480 nm, but also more significantly promote the intensity of red fluorescence at 616 nm (Figure 2b). The quantum yield results demonstrated the encapsulation of CB[7] increased the quantum yield of BPSP from 2.67% to 3.27% (Figure S28, Supporting Information), while the quantum yield of ring‐open BPMC was boosted from 1.04% to 2.18% (Figure S29, Supporting Information). According to the Ex‐Em mapping spectra, it could be found that the BPSP⊂CB[7] emitted steady fluorescence at 480 nm by the optimal excitation wavelength of 390 nm, whereas BPMC⊂CB[7] featured the same optimal excitation wavelength of 390 nm (Figure S30, Supporting Information). In addition, the assembly BPMC⊂CB[7] also showed good photo‐responsiveness, the absorption peak at 505 nm gradually decreased and finally disappeared after visible light irradiation for 30 s based on the UV‐vis absorption spectra. When the assembly were kept in the dark for a few hours, the reversible recovery efficiency of BPMC⊂CB[7] was calculated to be 97% (Figure S31, Supporting Information). In the fluorescence spectra, it was observed that the red fluorescence peak of BPMC⊂CB[7] at 616 nm gradually decreased under visible light irradiation, with a subsequent enhancement of the blue fluorescence peak at 480 nm (Figure 2c). According to the change of chromaticity coordinates depicted in the CIE 1931 chromaticity diagram, the fluorescence color of BPMC⊂CB[7] under visible light irradiation gradually shifted from an orange‐red to a blue (Figure 2e), which was in accordance with the photographs of the solution taken under a handheld UV lamp. Under dark conditions, the fluorescence emission was reversibly changed from blue to orange‐red due to the gradual conversion of BPSP⊂CB[7] to BPMC⊂CB[7] (Figure 2d), and the energy transfer efficiency from ring‐closed BPSP⊂CB[7] to the ring‐open BPMC⊂CB[7] was 86.08%. Reversible modulation in the fluorescence of the supramolecular system was achieved by alternating visible light irradiation and dark treatment, which were cycled for several times and showed good fatigue resistance (Figure 2f).

**Figure 3:**
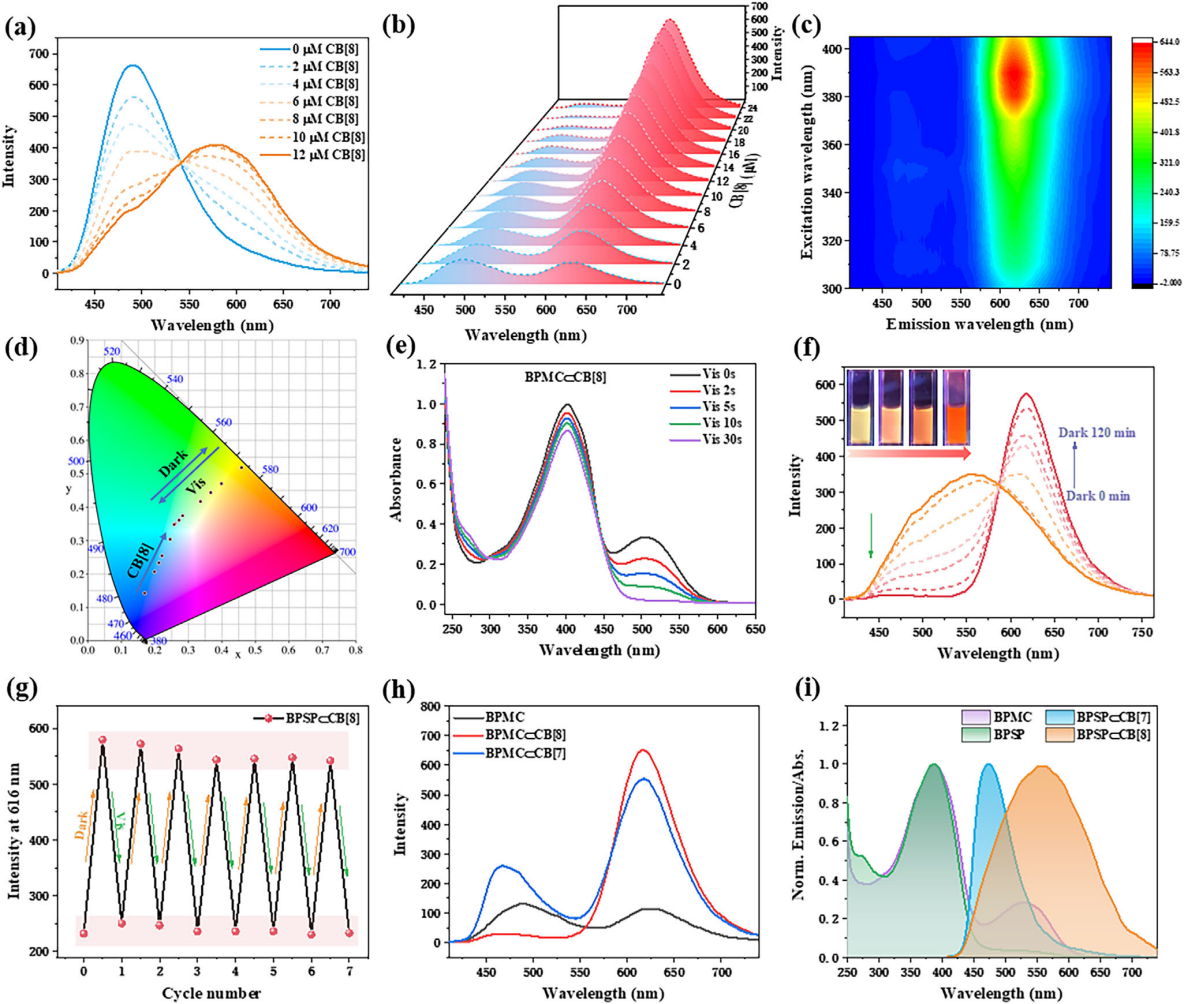
a) Fluorescence spectra of BPSP with the addition of CB[8] ([BPSP] = 2.0×10^−5^ M, [CB[8]] = 0–1.2 × 10^−5^ M). (b) Fluorescence spectra of BPMC with the addition of CB[8] ([BPMC] = 2.0 × 10^−5^ M, [CB[8]] = 0–2.4 × 10^−5^ M). c) Ex‐Em mapping spectra of BPMC⊂CB[8] upon visible light irradiation ([BPMC] = 2.0 × 10^−5^ M, [CB[8]] = 2.0 × 10^−5^ M). d) The CIE 1931 chromaticity diagram of BPSP with the addition of CB[8] and cyclic visible light as well as darkness. e) UV‐vis absorption spectra of BPSP⊂CB[8] upon visible light. f) Time‐dependent fluorescence spectra of BPSP⊂CB[8] in the dark. Inset: fluorescence image of BPSP⊂CB[8] under UV portable light. g) Fluorescence intensity at 616 nm of BPSP⊂CB[8] during cyclic darkness and visible irradiation ([BPSP] = 2.0 × 10^−5^ M, [CB[8]] = 2.0 × 10^−5^ M). h) Fluorescence spectra of BPMC, BPMC⊂CB, [7] and BPMC⊂CB[8] ([BPSP] = 2.0 × 10^−5^ M, [CB[7]] = [CB[8]] = 2.0 × 10^−5^ M). i) Normalized fluorescence spectra of BPSP⊂CB, [7] BPSP⊂CB[8] and absorption spectra of BPSP, BPMC ([BPSP] = [BPMC] = 2.0 × 10^−5^ M, [CB[7]] = [CB[8]] = 2.0 × 10^−5^ M).

In order to investigate the role of CB[8] on the fluorescence changes of guest molecules, fluorescence titration experiments were carried out. As shown in **Figure** **3**a, under visible light irradiation, increasing the concentration of CB[8] resulted in a decrease in the original blue fluorescence peak of BPSP at 480 nm, while a new fluorescence peak appeared at 570 nm. When the concentration of CB[8] reached 0.5 equivalents of BPSP, the fluorescence of the supramolecular system of BPSP⊂CB[8] no longer changed, and the quantum yield of yellow fluorescence emitted by BPSP⊂CB[8] was measured to be 2.81% (Figure S32, Supporting Information). The center of the fluorescence peak of BPSP⊂CB[8] at different excitation wavelengths was consistently ≈570 nm, and the optimal excitation wavelength was still 390 nm (Figure S33, Supporting Information). For the ring‐opened BPMC in a dark environment, a gradual decrease in the initial 480 nm fluorescence peak and an increase in the intensity of the 616 nm fluorescence peak were observed after the gradual addition of CB[8] to its aqueous solution (Figure 3b). Eventually, when the concentration ratio of CB[8] and BPMC was 1:1, the fluorescence emission of BPMC⊂CB[8] was completely transformed into a single red fluorescence at 616 nm. The quantum yield of BPMC⊂CB[8] (3.07%) was enhanced compared to BPMC (1.04%) (Figure S34, Supporting Information), suggesting the positive effect of CB[8] encapsulation of BPMC for promoting the intensity of red fluorescence. In the excitation wavelength range of 300–405 nm, BPMC⊂CB[8] exhibited a sole red fluorescence at 616 nm with the optimal excitation wavelength remaining at 390 nm (Figure 3c). Multiple fluorescent ranging from blue, cyan, yellow to red were obtained by increasing the proportion of CB[8] in the ring‐closed BPSP and the ring‐open BPMC, respectively, according to the variation of the chromatic coordinates tracked in the CIE 1931 diagram (Figure 3d). The responsiveness of the CB[8]‐mediated assembly to light stimuli was further explored. It was found the hypochromatic shift of BPMC⊂CB[8] fluorescence peak from 616 to 570 nm with visible light irradiation and reached equilibrium after 30 s of irradiation (Figure S35, Supporting Information). Meanwhile, UV‐vis absorption spectra showed that the absorption peak at 505 nm of BPMC⊂CB[8] disappeared after visible light irradiation, indicating the transition to the ring‐close BPSP⊂CB[8] (Figure 3e). In addition, after keeping in the dark for a few hours, the recovery efficiency of BPMC⊂CB[8] was calculated to be 89% according to the standard curve (Figure S36, Supporting Information). On the contrary, the fluorescence emission of the aqueous solution of BPSP⊂CB[8] was reversibly restored from yellow to red after keeping in the dark for 2 h, which was consistent with the change in the luminescence color of the solution as observed by the naked eye under UV light (Figure 3f). This reversible transformation process could be cycled more than six times under alternating visible light irradiation and dark treatment, exhibiting favorable fatigue resistance (Figure 3g; Figures S37 and S38, Supporting Information). Comparing the effects of CB[7] and CB[8] on the ring‐closed BPSP and ring‐open BPMC, it was found that different from the enhanced blue fluorescence caused by CB[7] encapsulated BPSP, CB[8] exhibited a red‐shifted yellow fluorescence emission for the encapsulation of BPSP (Figure S39, Supporting Information). The fluorescence spectra of BPMC⊂CB[8] exhibited a more complete transition from blue fluorescence of the *p*‐styrylpyridine moiety to red fluorescence of the spiropyran moiety in comparison to BPMC⊂CB[7] in the dark (Figure 3h). From Figure 3i, the fluorescence emission peak of BPSP⊂CB[7] was narrower and partially overlapped with the characteristic absorption peak at 540 nm of the spiropyran moiety in BPMC. The fluorescence peak of BPSP⊂CB[8] was wider and completely covered the absorption peak of BPMC at 540 nm, thereby, the incorporation of CB[8] was more conducive to promoting fluorescence resonance energy transfer (FRET) from the donor *p*‐styrylpyridine chromophore to the acceptor spiropyran luminophore than that of CB[7]. Thus, the photo‐controlled time‐dependent multicolor reversible luminescence was realized.

### 2.3 Applications in Logic Gates, Information Encryption and Tunable Cell Imaging

**Figure 4:**
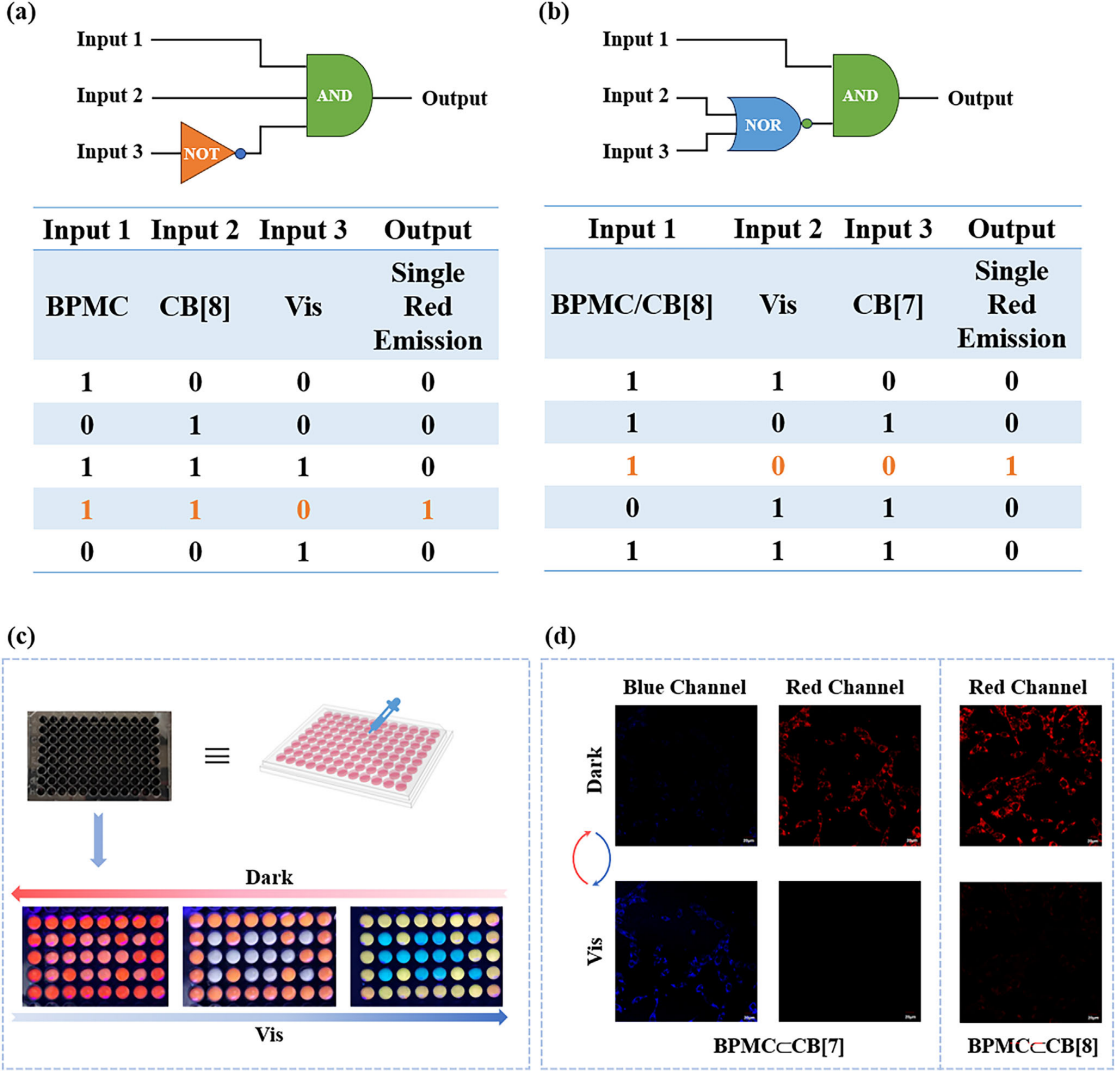
Schematic diagram and corresponding results for constructing the logic gate containing a) “NOT” gate and b) “NOR” gate. c) The process of dynamic information encryption and light‐driven decryption using BPMC⊂CB[8] and BPMC⊂CB.[7]. d) Confocal laser scanning microscopy images of photo‐modulation after co‐incubation of assemblies and A549 cells ([BPSP] = 2.0 × 10^−5^ M, [CB[8]] = 2.0 × 10^−5^ M, [CB[7]] = 2.0 × 10^−5^ M).

In view of the excellent light‐responsive multicolor luminescence properties of the supramolecular assemblies BPSP⊂CB[8] and BPSP⊂CB[7], they have shown potential applications in the construction of logic gates, encryption of dynamic information, and even tunable cellular imaging. As shown in **Figure** **4**, two logic gates were constructed taking a single red fluorescent emission at 616 nm of different assemblies as the output signal. The output signal was set to “1” for a single red fluorescence emission and “0” for no red fluorescence or two fluorescence emission peaks. When the inputs were BPMC and CB[8], the output of a single red fluorescence emission was “locked”. By using visible light irradiation as the “NOT” gate, no red fluorescence occurred and the output signal was “silent”. In the case of the coexistence of BPMC/CB[8], the output of single red fluorescence was “locked”. Both CB[7] and visible light irradiation as “NOR” gate inputs resulted in “silent” output signals. Detailed results were clearly presented, which illustrated the supramolecular system could realize a wide range of complex signal outputs using simple logic gates. Since the supramolecular assemblies BPMC⊂CB[8] and BPMC⊂CB[7] possessed distinct luminescent properties modulated by photo‐stimulation, they were able to be applied to information encryption. The aqueous solutions of BPMC⊂CB[8] and BPMC⊂CB[7] were added dropwise into 96‐well plates respectively. BPMC⊂CB[8] solution was poured into the edge of the 96‐well plate as the background, and the BPMC⊂CB[7] was emptied into specific wells to generate potential information. Initially, the background composed of BPMC⊂CB[8] and the encrypted message consisting of BPMC⊂CB[7] emitted similar red fluorescence, at which point the message was concealed. Upon the visible light irradiation, the English letters “NK” with blue fluorescence were gradually displayed, and the information could then be encrypted again under darkness, thus realizing dynamically adjustable information anti‐counterfeiting (Figure 4c). In addition, we also explored the controllable bioimaging of the two assemblies in A549 cells. As shown in Figure 4d, BPMC⊂CB[7] co‐incubated with A549 cells showed obvious luminescence in the red fluorescence channel under the laser confocal fluorescence microscope, while the blue channel could hardly capture any luminescence. After 30 s of visible light irradiation, the red fluorescence disappeared. On the contrary, the corresponding fluorescence image could be detected in the blue fluorescence channel. On the other hand, A549 cells produced red fluorescence imaging after co‐incubation with BPMC⊂CB [8], and visible light irradiation caused the image of the red fluorescence channel to almost disappear, demonstrating the potential of both assemblies in tunable cell imaging.

## 3 Conclusion

In conclusion, we constructed two different photo‐controlled time‐dependent bidirectional multicolor luminescent supramolecular shuttles BPSP⊂CB[8] and BPSP⊂CB[7]. Specifically, CB[7] encapsulated BPSP displaying macrocycle confinement and effectively enhanced the fluorescence intensity of visible‐light‐induced ring‐open BPMC and ring‐closed BPSP in the dark, achieving reversible multicolor luminescence from blue to orange. At the same time, CB[7] encapsulated guest molecules to form regular hexagonal nanosheets. Different from BPSP⊂CB [7], CB[8] bonded ring‐closed BPSP at a ratio of 1:2 and induced the luminescent bathochromic shift from blue to yellow under visible light irradiation. In the dark environment, BPMC⊂CB[8] exhibited almost complete conversion from yellow fluorescence to red fluorescence at 616 nm over time due to the effective intramolecular energy transfer. It not only regulated topology morphology changes between small‐size nanoparticles and larger nanospheres due to the change of assembly mode but also realized different multicolor luminescence. Two different multicolor luminescent supramolecular bidirectional shuttles driven by light have been successfully applied to construct logic gates, dynamic information encryption, and tunable cell imaging, which provided a new approach for the construction of tunable luminescence and its multiple applications.

## Conflict of Interest

The authors declare no conflict of interest.

## Supporting information



Supporting Information

## Data Availability

The data that support the findings of this study are available from the corresponding author upon reasonable request.

## References

[1] X. Long , Z. Ma , H. Dai , Y. Wang , H. Xie , X. Ge , Z. Yang , J. Zhao , W. Hong , Z. Chi , Aggregate 2025, 6, 70006.

[2] M. Tang , J. Wen , Y. Sun , Q. Hou , X. Cai , W. He , X. Xie , H. Ding , F. Li , L. Zheng , Y. Shi , Q. Cao , Adv. Funct. Mater. 2024, 34, 2314130.

[3] Y.‐G. Wu , W.‐L. Zhou , Y. Qiu , S. Wang , J. Liu , Y. Chen , X. Xu , Y. Liu , Adv. Sci. 2025, 2415418. 10.1002/advs.202415418 PMC11984880 39950854

[4] Z. Ping , F. Xie , X. Gong , F. Zhang , J. Zheng , Y. Liu , J. Leng , Adv. Funct. Mater. 2024, 34, 2402592.

[5] Y. Gao , Y. Yang , Y. Wei , Y. Li , H. Cai , C. Wu , Adv. Funct. Mater. 2025, 35, 2416025.

[6] Y. Pei , Y. Fan , K. Sun , D. Hu , Y. Liu , J. Yin , L. Chen , M. Xu , W. Yan , X. Liu , F. Li , Angew. Chem., Int. Ed. 2025, 137, 202423791. 10.1002/anie.202423791 39895363

[7] Y. Zhao , Y. Mei , J. Sun , Y. Tian , J. Am. Chem. Soc. 2025, 147, 5025. 39882873 10.1021/jacs.4c14727

[8] J. Yu , H. Yu , Y. Qiu , H.‐Y. Zhang , X. Xu , Y. Liu , Angew. Chem., Int. Ed. 2025, 64, 202418938. 10.1002/anie.202418938 39513650

[9] Y. He , Y. Qiao , Z. Li , W. Feng , Y. Zhao , W. Tian , B. Zhong Tang , H. Yan , Angew. Chem., Int. Ed. 2024, 63, 202413425. 10.1002/anie.202413425 39136193

[10] F. Hassan , Y. Tang , H. K. Bisoyi , Q. Li , Adv. Mater. 2024, 36, 2401912. 10.1002/adma.202401912 38847224

[11] M. Bayat , H. Mardani , H. Roghani‐Mamaqani , R. Hoogenboom , Chem. Soc. Rev. 2024, 53, 4045. 38449438 10.1039/d3cs00431g

[12] J. Tang , Y. Ren , J. Feng , Chem. Eng. J. 2022, 440, 135932.

[13] H. Zhao , S. Hu , M. Liang , P. Xue , Adv. Opt. Mater. 2025, 13, 2402321.

[14] A. Schulz , R. Fröhlich , A. Jayachandran , F. Schneider , M. Stolte , T. Brixner , F. Würthner , Chem 2024, 10, 2887.

[15] X. Chen , F.‐Y. Chen , Y. Lu , Q. Li , S. Li , C. Zheng , Y. Zheng , L. Dang , R.‐Y. Li , Y. Liu , D.‐S. Guo , S.‐K. Sun , Z. Zhang , Adv. Sci. 2024, 11, 2404731. 10.1002/advs.202404731 PMC11423228 39072943

[16] Z. Yang , Y. Wang , X. Liu , R. T. Vanderlinden , R. Ni , X. Li , P. J. Stang , J. Am. Chem. Soc. 2020, 142, 13689. 32786812 10.1021/jacs.0c06666

[17] Z. He , J. Song , C. Li , Z. Huang , W. Liu , X. Ma , Adv. Mater. 2025, 37, 2418506. 10.1002/adma.202418506 39930926

[18] Y. Sun , X. Cai , W. He , X. Ji , L. Zheng , Y. Shi , Q. Cao , Adv. Opt. Mater. 2024, 12, 2401445.

[19] Y.‐Y. Ren , B.‐Y. Deng , Z.‐H. Liao , Z.‐R. Zhou , C.‐H. Tung , L.‐Z. Wu , F. Wang , Adv. Mater. 2023, 35, 2307971. 10.1002/adma.202307971 37743568

[20] J. Rühe , K. Vinod , H. Hoh , K. Shoyama , M. Hariharan , F. Würthner , J. Am. Chem. Soc. 2024, 146, 28222. 10.1021/jacs.4c08479 39264316

[21] H. Cheng , T. Liu , J. Tian , R. An , Y. Shen , M. Liu , Z. Yao , Adv. Sci. 2024, 11, 2309259. 10.1002/advs.202309259 PMC11267353 38760900

[22] G. Zhang , F. Chen , Y. Di , S. Yuan , Y. Zhang , X. Quan , Y. Chen , H. Chen , M. Lin , Adv. Funct. Mater. 2024, 34, 2404123.

[23] S. Garain , K. Shoyama , L.‐M. Ginder , M. Sárosi , F. Würthner , J. Am. Chem. Soc. 2024, 146, 22056. 39047068 10.1021/jacs.4c07730 PMC11311229

[24] J. Pan , W. Lin , F. Bao , Q. Qiao , G. Zhang , Y. Lu , Z. Xu , Chin. Chem. Lett. 2023, 34, 107519.

[25] T. Ma , T. Li , L. Zhou , X. Ma , J. Yin , X. Jiang , Nat. Commun. 2020, 11, 1811. 32286298 10.1038/s41467-020-15600-6 PMC7156701

[26] G. Wu , J.‐R. Wu , Y. Wang , Y.‐W. Yang , Chem 2023, 9, 2918.

[27] H. Xu , Q. Wang , Z. Qi , X. Li , Y. Lei , H. An , H. Tian , D.‐H. Qu , Angew. Chem. Int. Ed. 2025, 64, 202420707. 10.1002/anie.202420707 39617729

[28] M. Tian , Z. Wang , X. Yuan , H. Zhang , Z. Liu , Y. Liu , Adv. Funct. Mater. 2023, 33, 2300779.

[29] L. Hu , Y. Gao , Q. Cai , Y. Wei , J. Zhu , W. Wu , Y. Yang , J. Colloid Interface Sci. 2024, 665, 545. 38547635 10.1016/j.jcis.2024.03.129

[30] K. Wang , X. Ou , X. Niu , Z. Wang , F. Song , X. Dong , W.‐j. Guo , H.‐Q. Peng , Z. Zhao , J. W. Y. Lam , J. Sun , H. Wu , S.‐Y. Yu , F. Li , B. Z. Tang , Aggregate 2025, 6, 667.

[31] M. Babazadeh‐Mamaqani , D. Razzaghi , H. Roghani‐Mamaqani , A. Babaie , M. Rezaei , R. Hoogenboom , M. Salami‐Kalajahi , Prog. Mater. Sci. 2024, 146, 101312.

[32] Z. Meng , S. Taneja , R. Hassan , J. R. Parquette , ACS Appl. Mater. Interfaces 2024, 16, 47089. 39197171 10.1021/acsami.4c07280

[33] H. Cai , L. Hu , Y. Gao , C. Wu , Y. Wei , Y. Yang , Chem. Eng. J. 2024, 500, 157148.

[34] R. Zhang , Y. Chen , Y. Liu , Angew. Chem., Int. Ed. 2023, 62, 202315749. 10.1002/anie.202315749 37971202

[35] W. Wang , J. Dai , Z. Zhang , J. Zhang , H. Tian , J. Am. Chem. Soc. 2025, 147, 5486. 39879537 10.1021/jacs.4c18682

