# Supplementary material for: Multicolor Luminescent Supramolecular Bidirectional Shuttles Driven by Light
Source: Adv Sci (Weinh). 2025 Jun 4;12(32):e07090. doi: 10.1002/advs.202507090 (PMC12407255; doi:10.1002/advs.202507090)
Supplement: Supplementary file 1 — Supporting Information [file ADVS-12-e07090-s001.docx]

Supporting Information for

**Multicolor Luminescent Supramolecular Bidirectional Shuttle Driven by Light**

*Rong Zhang^[a]^, Zhuo Lei^[a]^, Zhiyi Yu^[a]^, Yong Chen^[a]^, Yu Liu*^[a]^*

*[a] College of Chemistry, State Key Laboratory of Elemento-Organic Chemistry, Nankai University, Tianjin 300071, China*

* Corresponding author:

E-mail: yuliu@nankai.edu.cn

**Experimental section**

**1. Instruments and Methods**

All reagents and solvents were commercially available and used without further purification unless otherwise noted. ^1^H NMR (400 MHz) and ^13^C NMR (100 MHz) spectra were investigated on Bruker Avance spectrometers. Mass spectra were performed on Varian 7.0T FTMS. UV-Vis data were collected on Shimadzu UV-3600 spectrophotometer (light path 10 mm, quartz cell). The fluorescence spectrum was collected on JASCO FP-750 spectrometer. Fluorescence lifetime and quantum efficiency were obtained on FLS5 instrument (Edinburg Instruments, Livingstone, UK). Transmission electron microscope (TEM) measurements were recorded on a high-resolution TEM (Tecnai G2 F20 microscope, FEI) equipped with a CCD camera (Orius 832, Gatan). Scanning electron microscope (SEM) images were measured on Apreo S LoVac instrument. The irradiation experiment was carried out at 3 cm from a 530 nm lamp (6W). Olympus FV1000 Laser scanning confocal microscope and Leika S8 microscope were applied to observe the cell images. All measurements were carried out at room temperature (RT) except for specifying otherwise.

**Sample preparations**: All sample preparations were dissolved in deionized water (with fw=1% DMSO) unless otherwise noted.

**2. Synthetic Protocols**

**
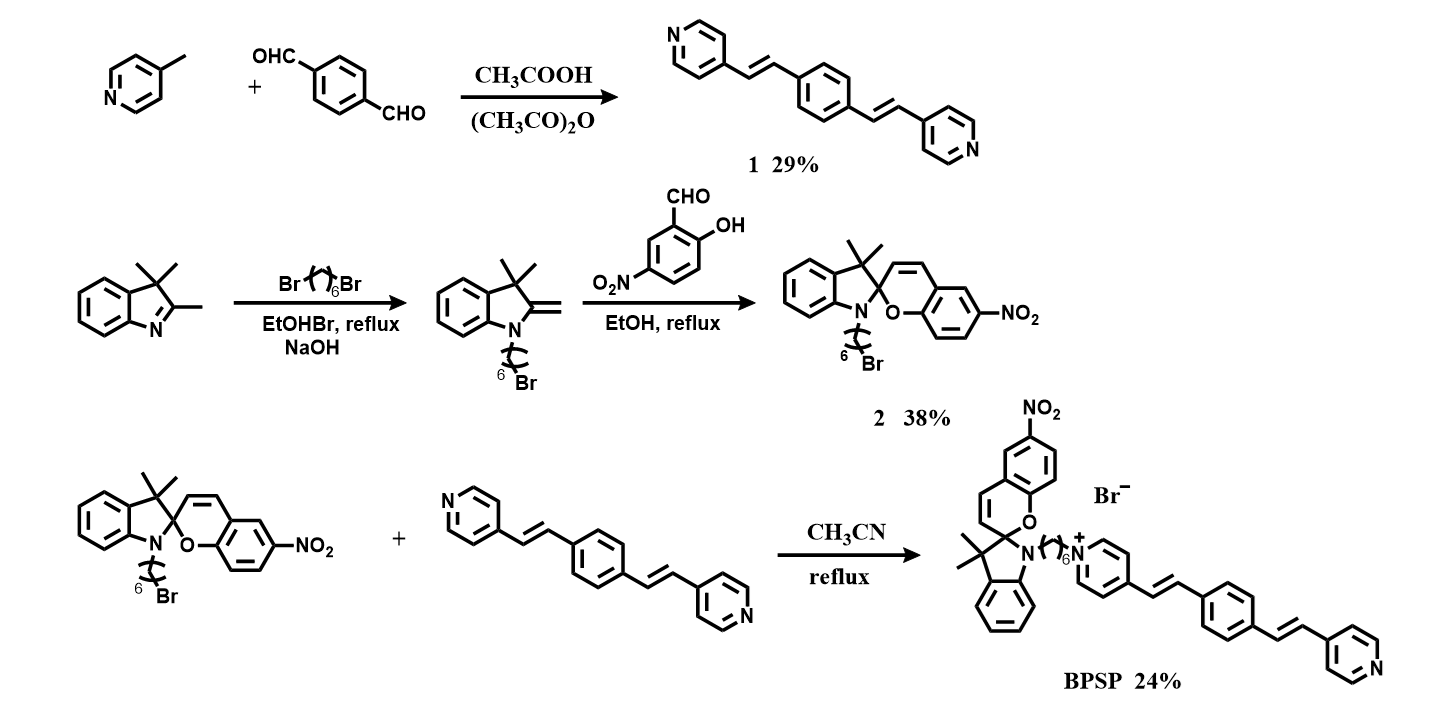
**

Scheme S1 Synthesis routes of BPSP.

**Synthesis of 1 and 2.** Compounds **1** and **2** were synthesized according to published literature^1, 2^.

**Synthesis of BPSP.** Compound **1** (0.34 mmol, 160 mg) and **2** (0.34 mmol, 100 mg) were dissolved in 40 mL CH_3_CN, and the reaction mixture was stirred and refluxed overnight under N_2_ atmosphere. After the reaction was complete, the reaction mixture was cooled to room temperature, solvent was removed under reduced pressure. Dissolve the residue with a small amount of CH_2_Cl_2_, drop it into ethyl acetate to precipitate, and filter to obtain a black solid which was further purified by column chromatography (CH_2_Cl_2_/CH_3_OH = 20 : 1). Finally, the product as an orange-red solid was obtained (yield: 20%). ^1^H NMR (400 MHz, DMSO-*d_6_*) δ [ppm]: 8.92 (d, *J*=6.8, 2H), 8.58 (d, *J*=6.0, 2H), 8.22 (d, *J*=13.3, 2H), 8.03 (d, *J*=16.3, 1H), 7.97 (d, *J*=11.8, 1H), 7.80 (s, 4H), 7.59 (t, *J*=15.0, 4H), 7.39 (d, *J*=16.5, 1H), 7.19 (d, *J*=10.4, 1H), 7.11 (t, *J*=6.2, 2H), 6.85 (d, *J*=9.0, 1H), 6.78 (t, *J*=7.2, 1H), 6.58 (d, *J*=7.9, 1H), 5.98 (d, *J*=10.4, 1H), 5.75 (s, 1H), 4.45 (t, *J*=7.2, 2H), 3.12 (d, *J*=8.4, 2H), 1.93-1.80 (m, 2H), 1.25 (m, 12H). ^13^C NMR (100 MHz, DMSO-*d_6_*) δ [ppm]: 159.17, 146.68, 144.26, 140.42, 135.61, 128.68, 128.00, 127.58, 125.69, 123.95, 123.66, 122.79, 121.73, 121.32, 118.93, 118.71, 115.43, 106.44, 52.23, 42.76, 30.46, 28.16, 25.94, 25.29, 19.50. HRMS (ESI) m/z for C_44_H_43_BrN_4_O_3_ calcd [M−Br]^+^: 675.3330, found: 675.3333.

**
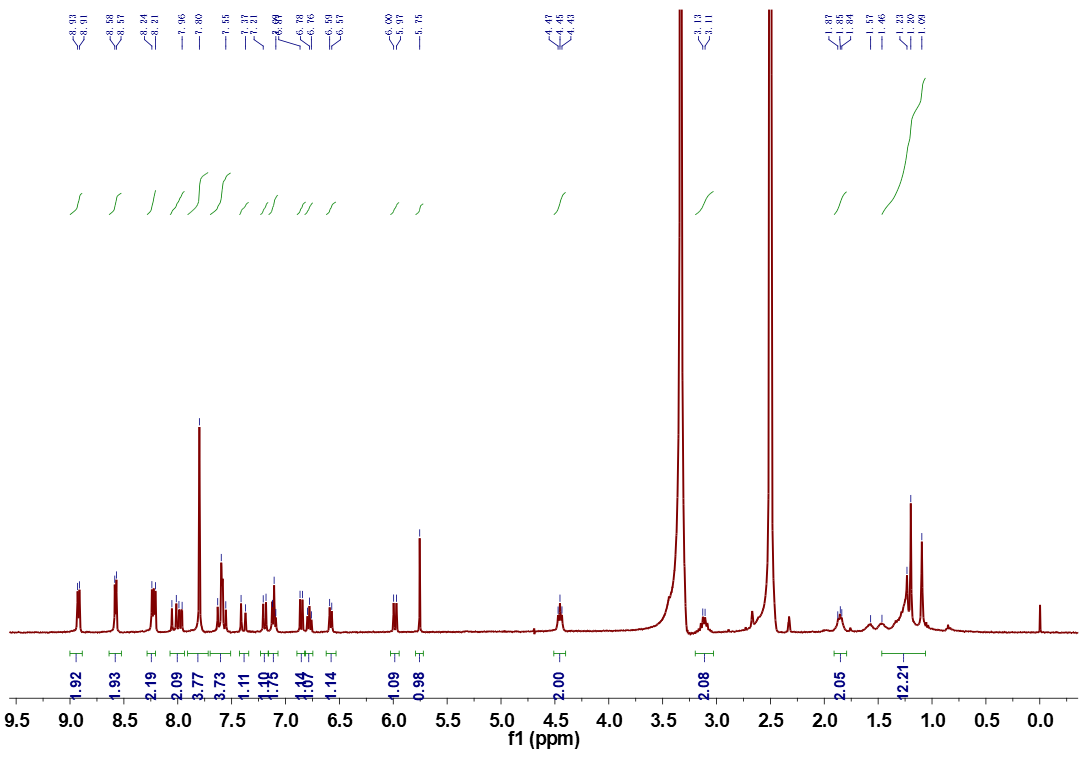
**

Figure S1. ^1^H NMR spectra of BPSP (400 MHz, DMSO-*d_6_*, 25 ℃).

**
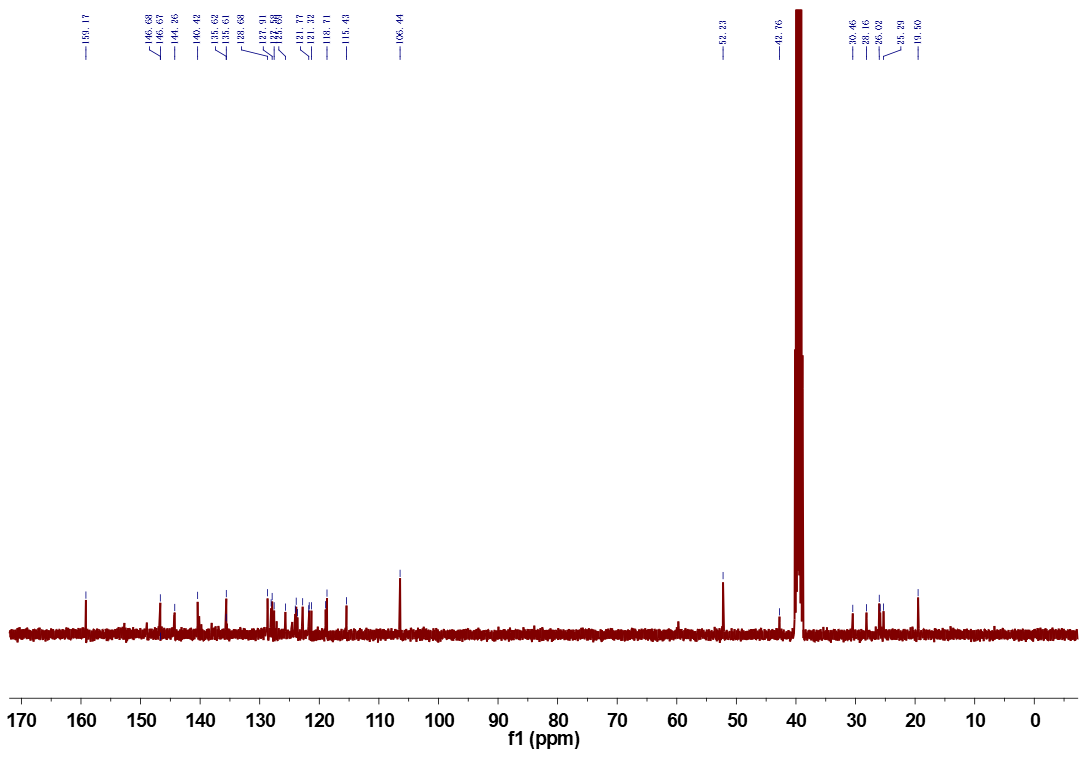
**

Figure S2. ^13^C NMR spectra of BPSP (100 MHz, DMSO-*d_6_*, 25 ℃).

**
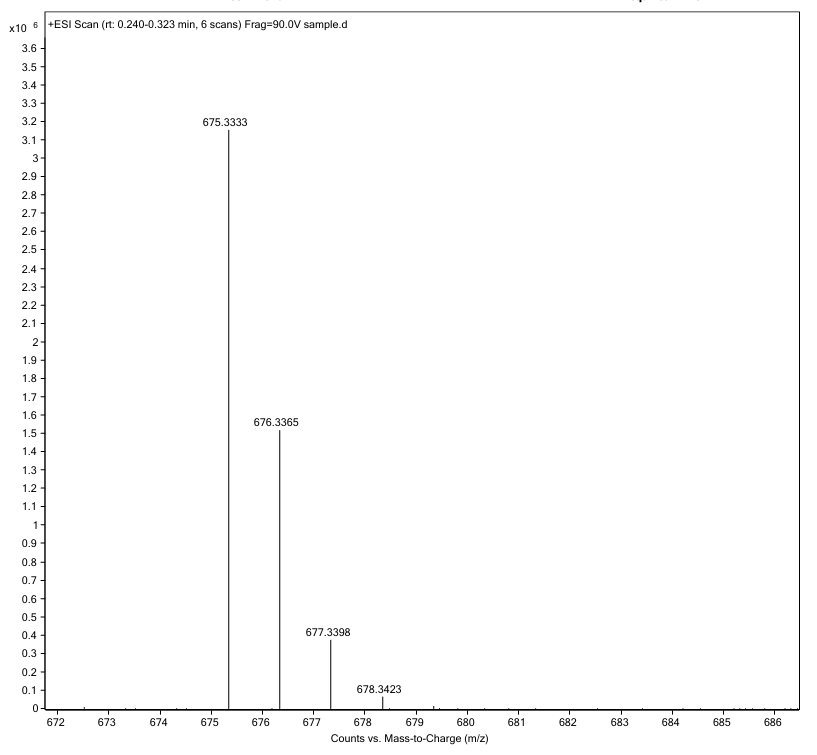
**

Figure S3. HRMS spectra of BPSP.

**3. Investigation of host-guest properties between BPSP and CB[7] or CB[8]**

**
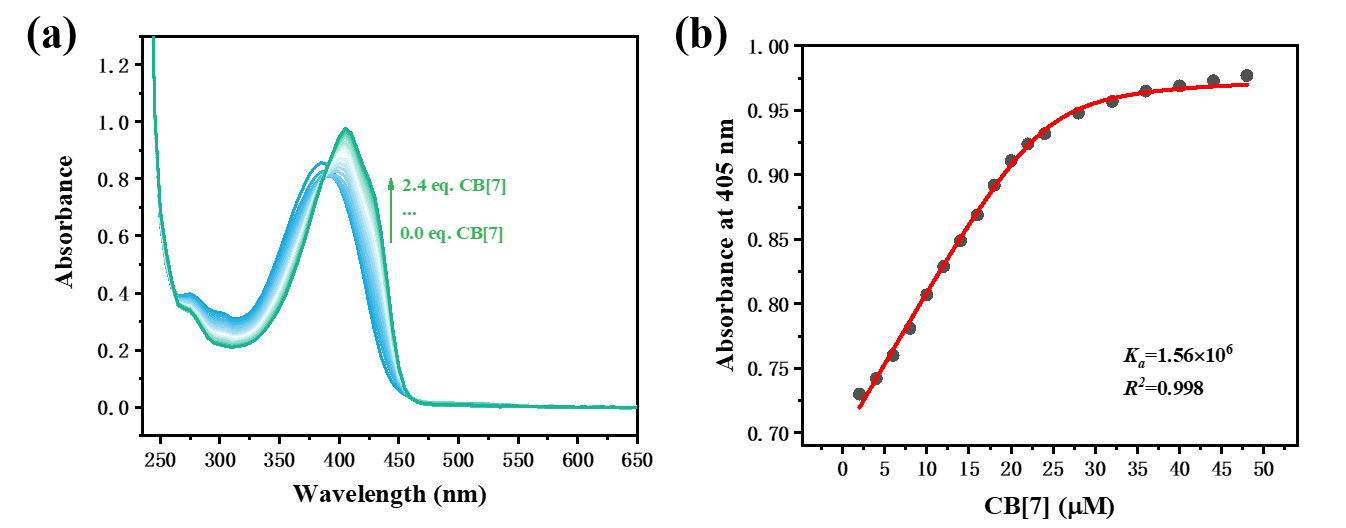
**

Figure S4. (a) UV-Vis absorption spectra of BPSP with the addition of CB[7] ([BPSP] = 2.0×10^-5^ M, [CB[7]] = 0-4.8×10^-5^ M). (b) The association constant (*K*_a_) of BPSP⊂CB[7] complexation ([BPSP] = 2.0×10^-5^ M, [CB[7]] = 0-4.8×10^-5^ M).

**
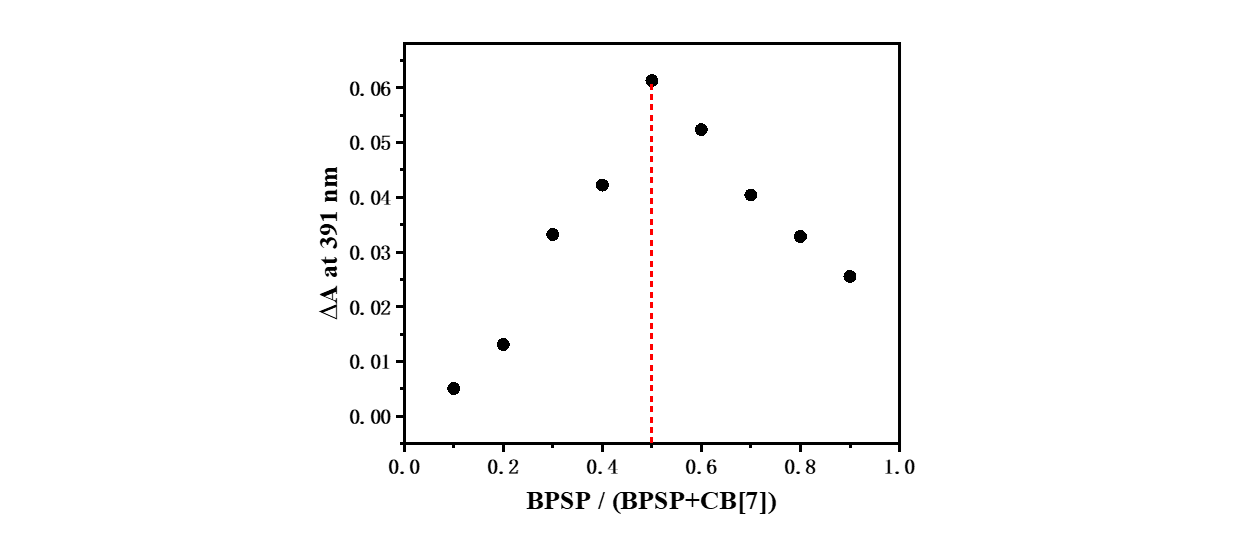
**

Figure S5. Job’s plot of BPSP⊂CB[7] ([BPSP] + [CB[7]] = 2.0×10^-5^ M).


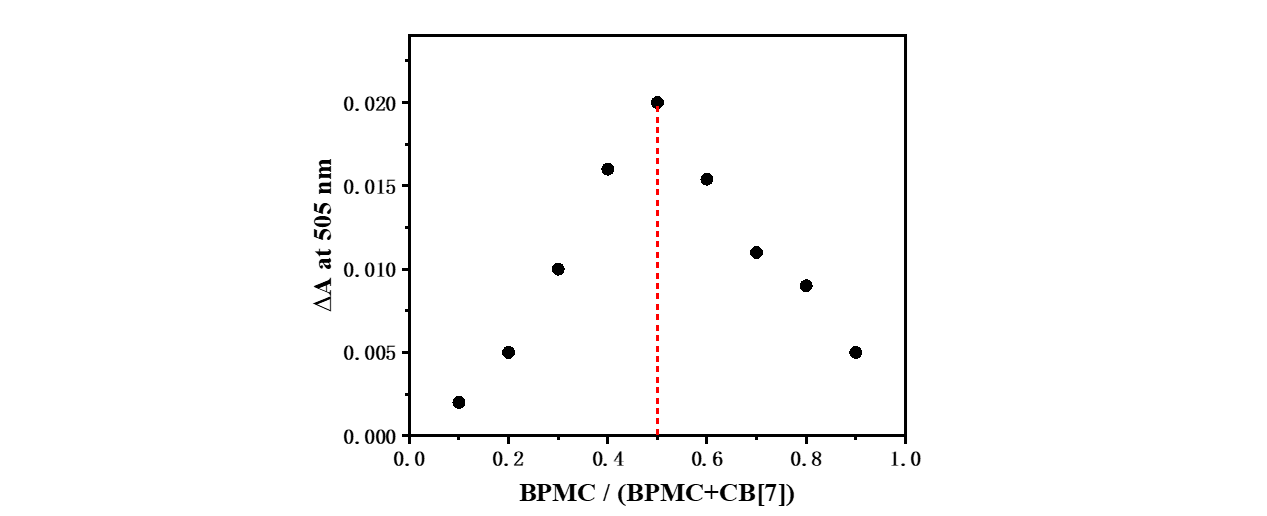


Figure S6. Job’s plot of BPMC⊂CB[7] ([BPMC] + [CB[7]] = 2.0×10^-5^ M).


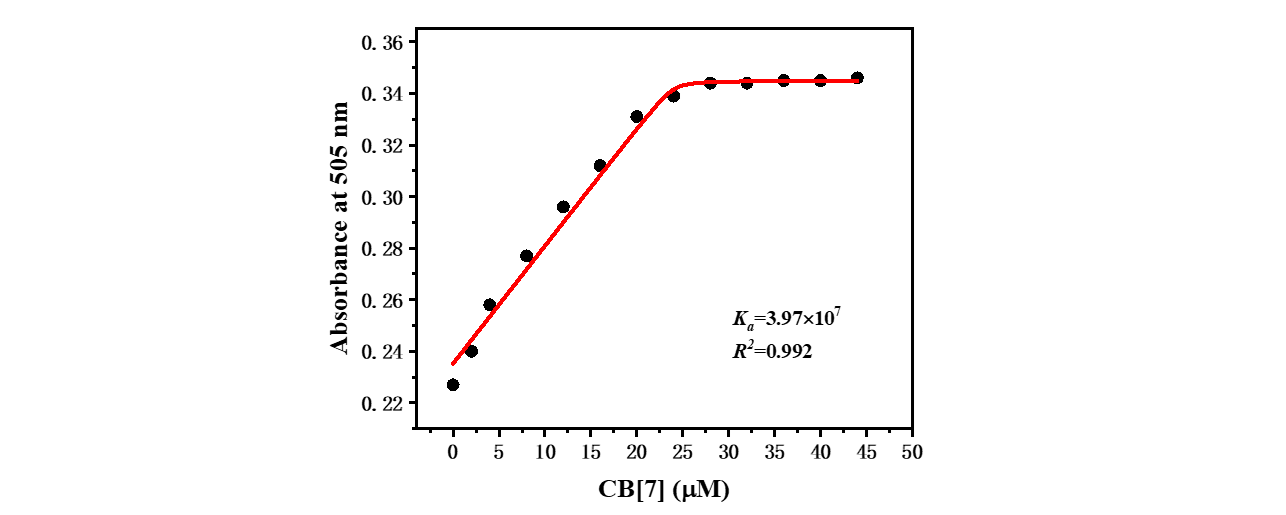


Figure S7. The association constant (*K*_a_) of BPMC⊂CB[7] complexation ([BPMC] = 2.0×10^-5^ M, [CB[7]] = 0-4.4×10^-5^ M).


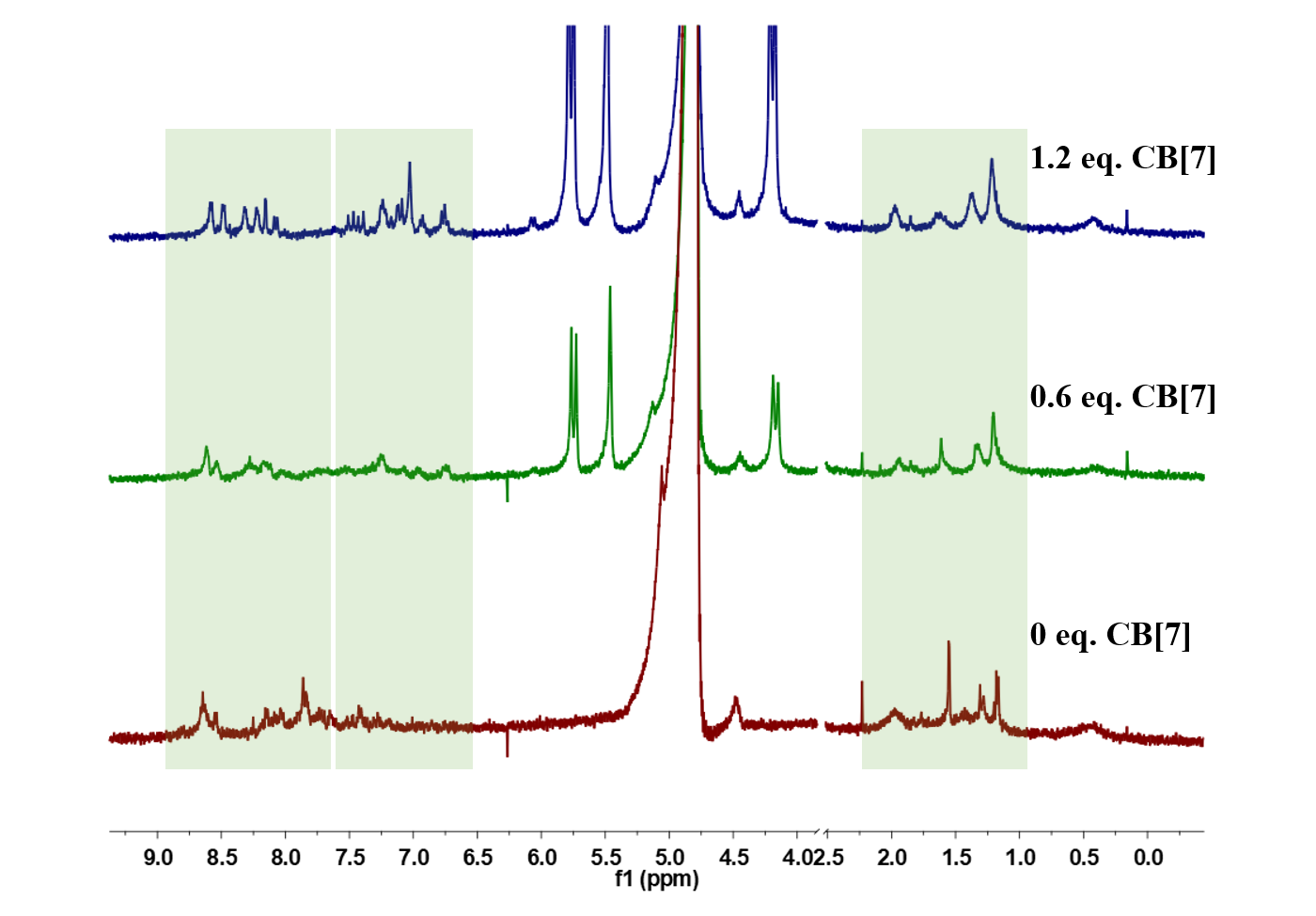


Figure S8. ^1^H NMR spectra of BPSP with the addition of CB[7] (400 MHz, D_2_O: DMSO-*d_6_* = 20:1, 25 ℃, [BPSP] = 5.0×10^-4^ M，[CB[7]] = 0, 3.0×10^-4^, 6.0×10^-4^ M)

Figure S9. MALDI-TOF mass spectra of BPMC⊂CB[7].


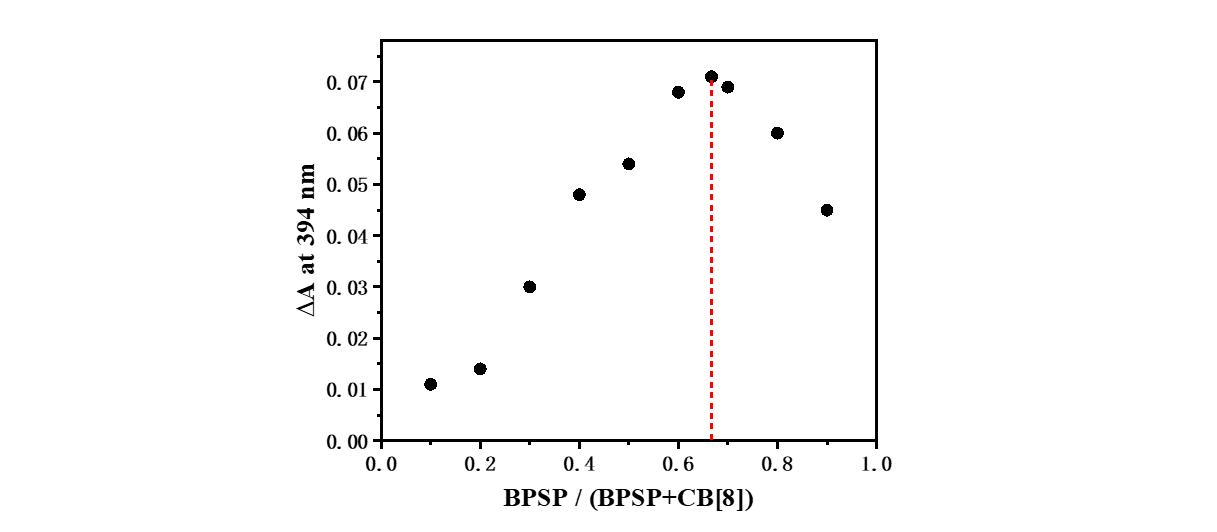


Figure S10. Job’s plot of BPSP⊂CB[7] ([BPSP] + [CB[7]] = 2.0×10^-5^ M).


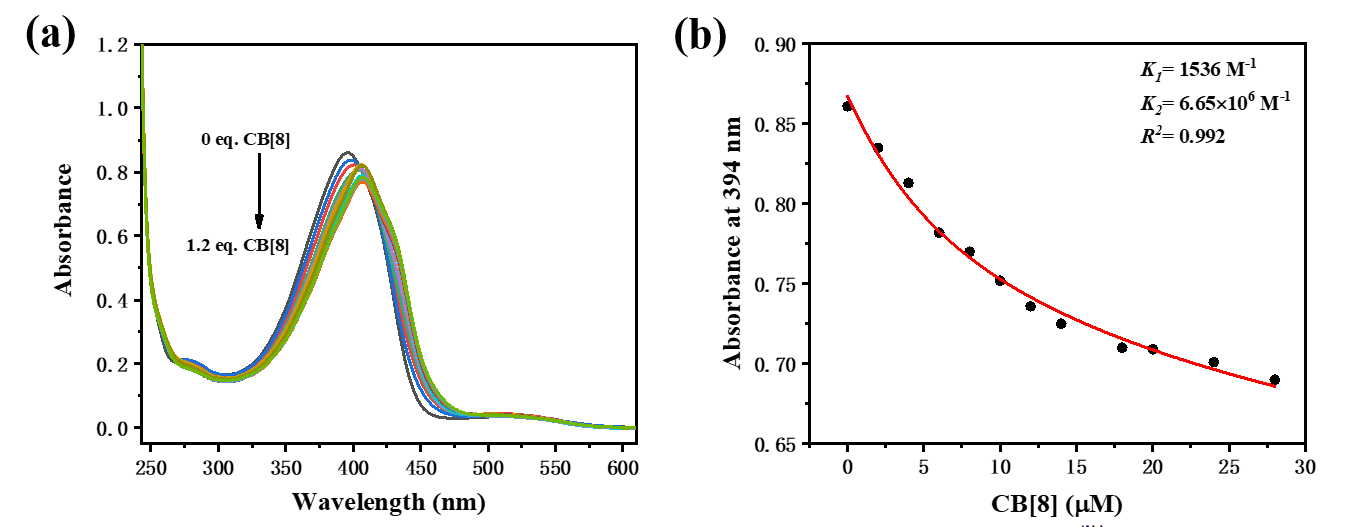


Figure S11. (a) UV-Vis absorption spectra of BPSP with the addition of CB[8] ([BPSP] = 2.0×10^-5^ M, [CB[8]] = 0-2.4×10^-5^ M). (b) The association constant (*K*_a_) of BPSP⊂CB[8] complexation ([BPSP] = 2.0×10^-5^ M, [CB[8]] = 0-4.4×10^-5^ M).


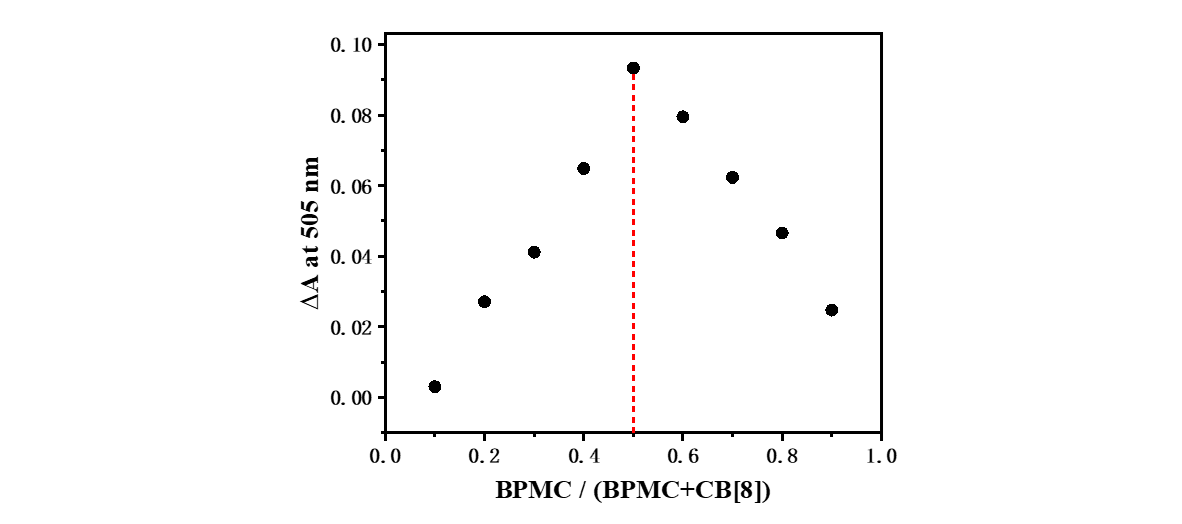


Figure S12. Job’s plot of BPMC⊂CB[8] ([BPMC] + [CB[8]] = 2.0×10^-5^ M).


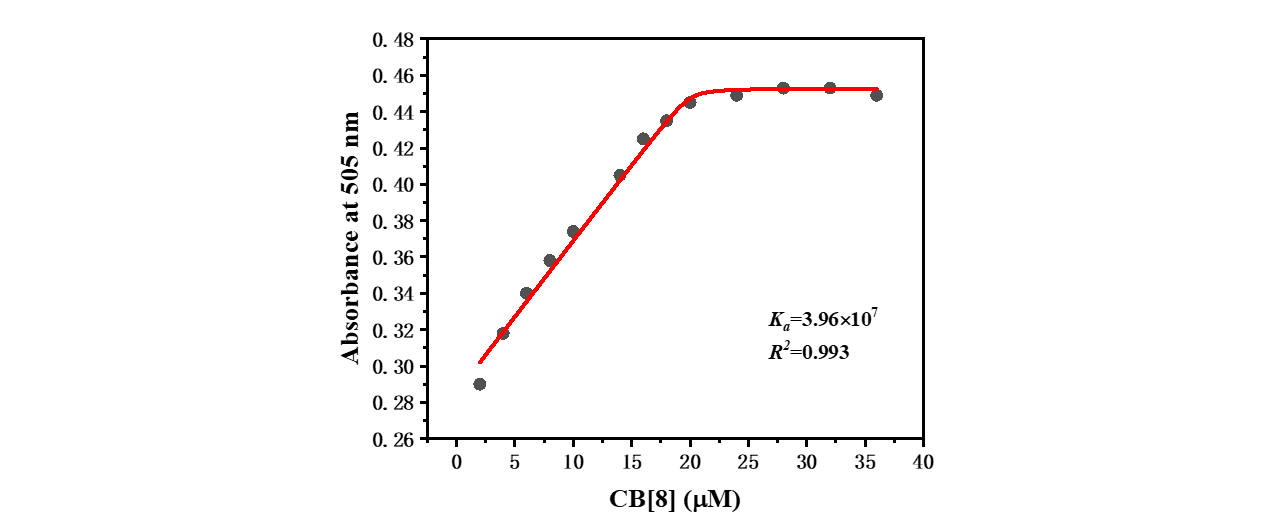


Figure S13. The association constant (*K*_a_) of BPMC⊂CB[8] complexation ([BPMC] = 2.0×10^-5^ M, [CB[8]] = 0-2.4×10^-5^ M).


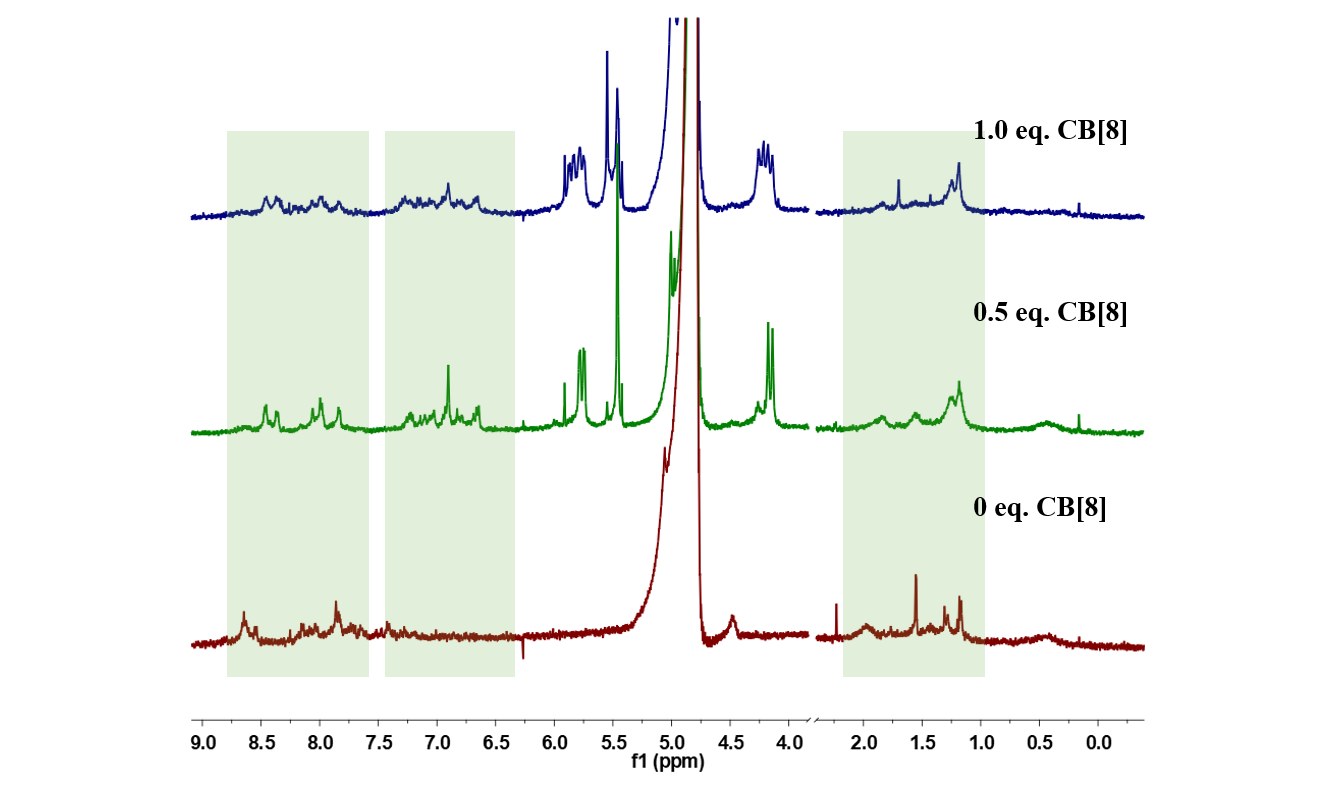


Figure S14. ^1^H NMR spectra of BPSP with the addition of CB[8] (400 MHz, D_2_O: DMSO-*d_6_* = 20:1, 25 ℃, [BPSP] = 5.0×10^-4^ M，[CB[8]] = 0, 2.5×10^-4^, 5.0×10^-4^ M)


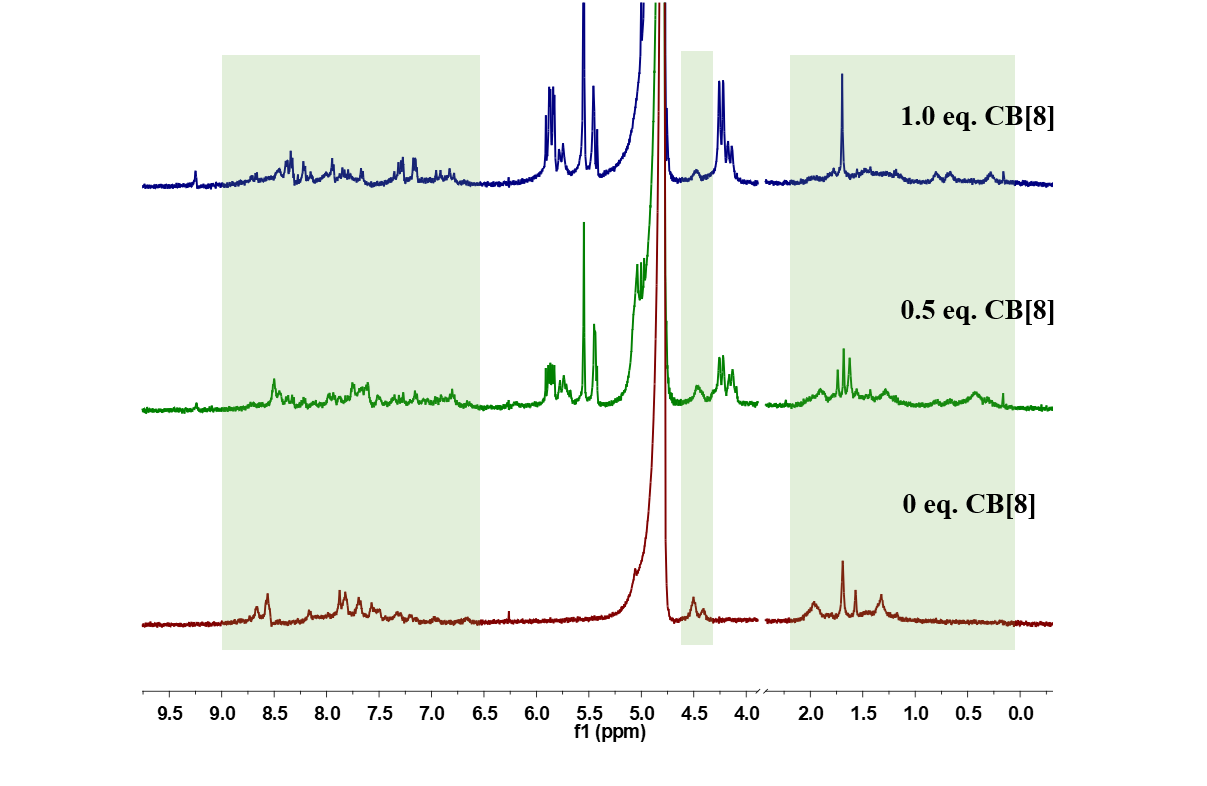


Figure S15. ^1^H NMR spectra of BPMC with the addition of CB[8] (400 MHz, D_2_O: DMSO-*d_6_* = 20:1, 25 ℃, [BPMC] = 5.0×10^-4^ M，[CB[8]] = 0, 2.5×10^-4^, 5.0×10^-4^ M)


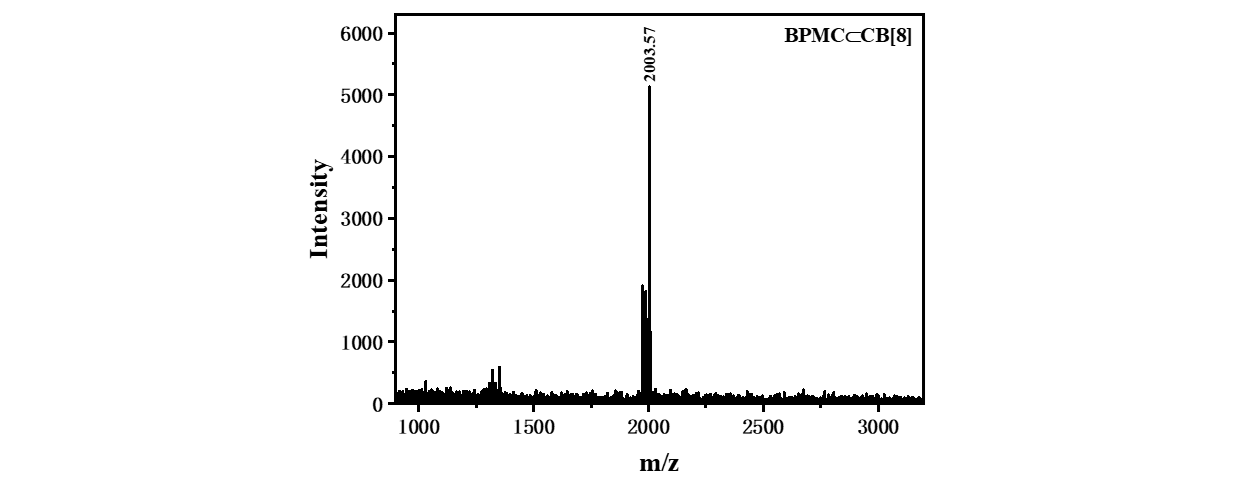


Figure S16. MALDI-TOF mass spectra of BPMC⊂CB[8].


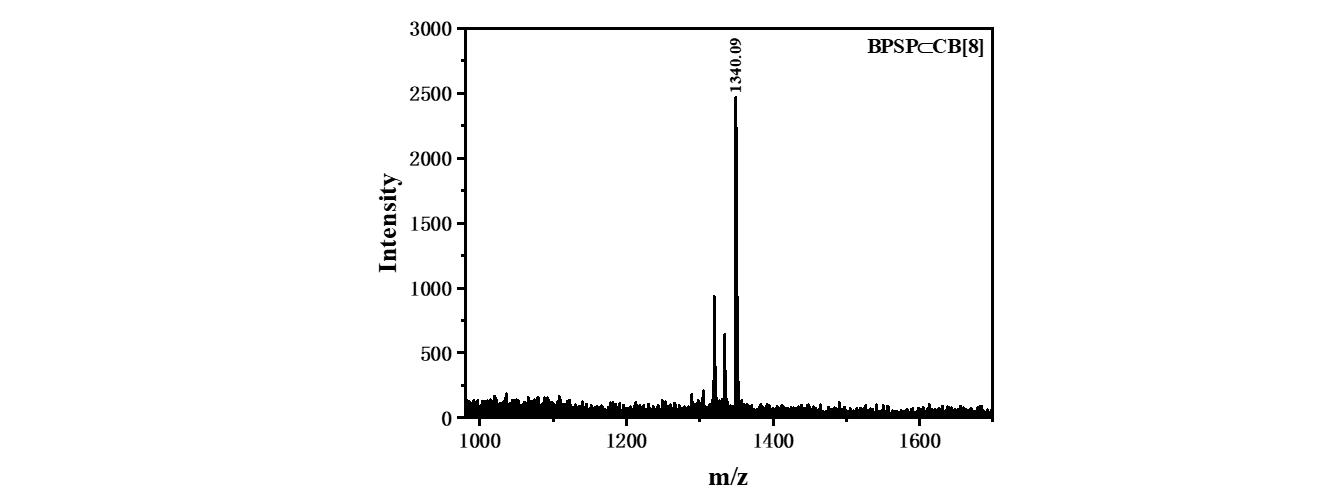


Figure S17. MALDI-TOF mass spectra of BPSP⊂CB[8].


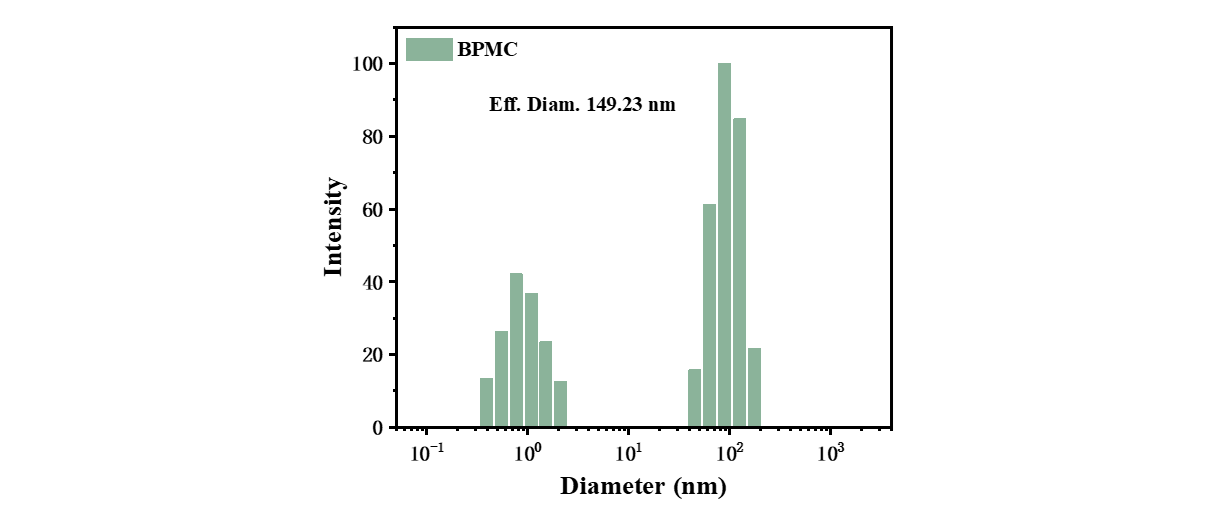


Figure S18. DLS results of BPMC ([BPMC] = 2.0×10^-5^ M).


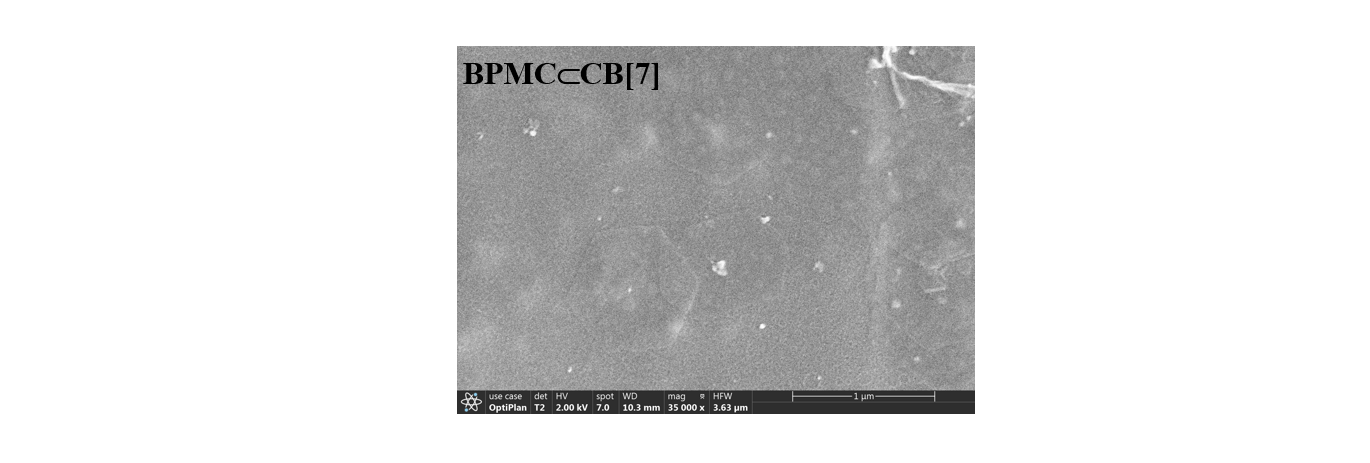


Figure S19. SEM image of BPMC⊂CB[7] ([BPMC] = 2.0×10^-5^ M, [CB[7]] = 2.0×10^-5^ M).


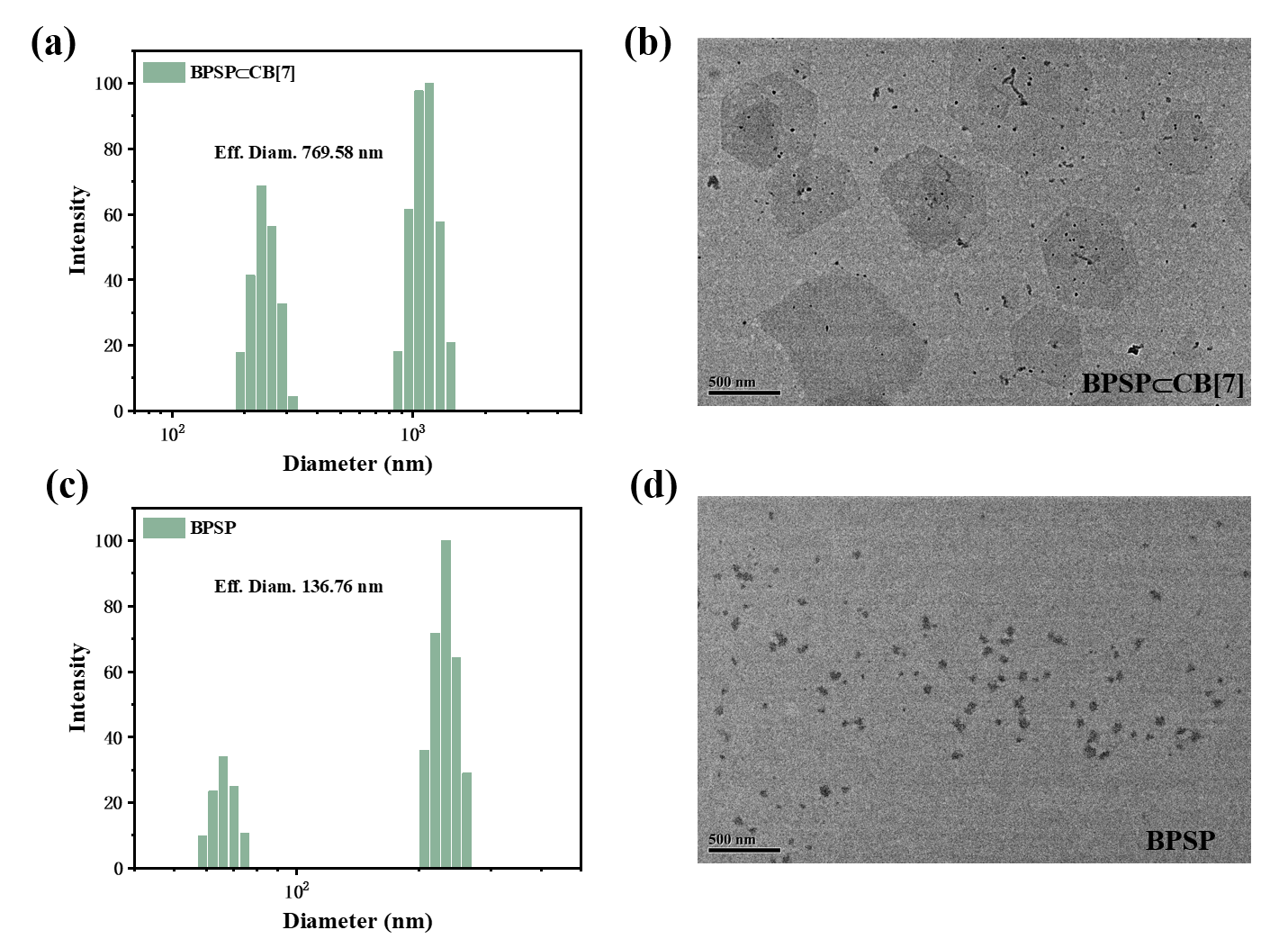
Figure S20. (a) DLS results of BPSP⊂CB[7]. (b) TEM image of BPSP⊂CB[7]. (c) DLS results of BPSP. (d) TEM image of BPSP ([BPSP] = 2.0×10^-5^ M, [CB[7]] = 2.0×10^-5^ M).


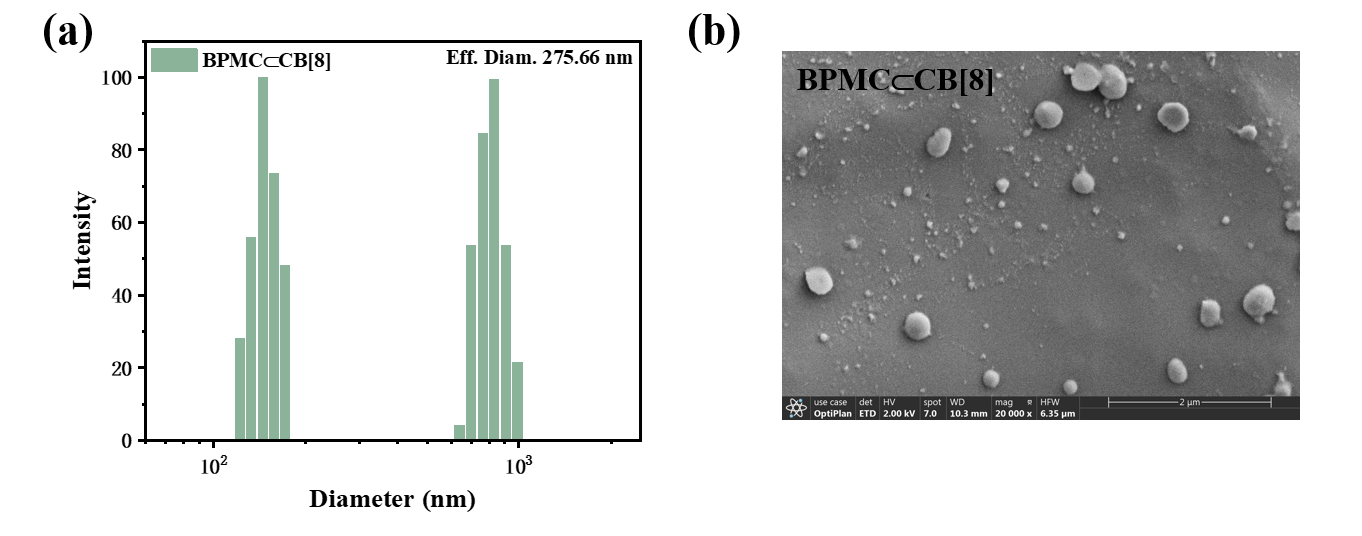


Figure S21. (a) DLS results of BPMC⊂CB[8]. (b) SEM image of BPMC⊂CB[8] ([BPMC] = 2.0×10^-5^ M, [CB[8]] = 2.0×10^-5^ M).


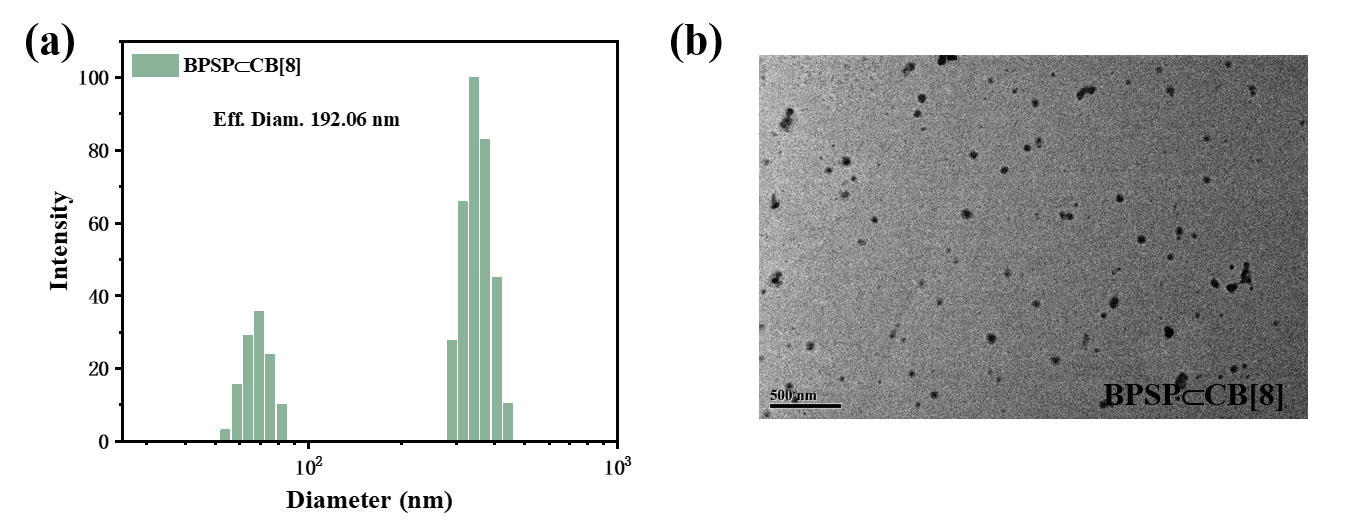


Figure S22. (a) DLS results of BPSP⊂CB[8]. (b) TEM image of BPSP⊂CB[8] ([BPSP] = 2.0×10^-5^ M, [CB[8]] = 2.0×10^-5^ M).

**4. Study on the photo-responsive properties of guest molecule BPSP**

**
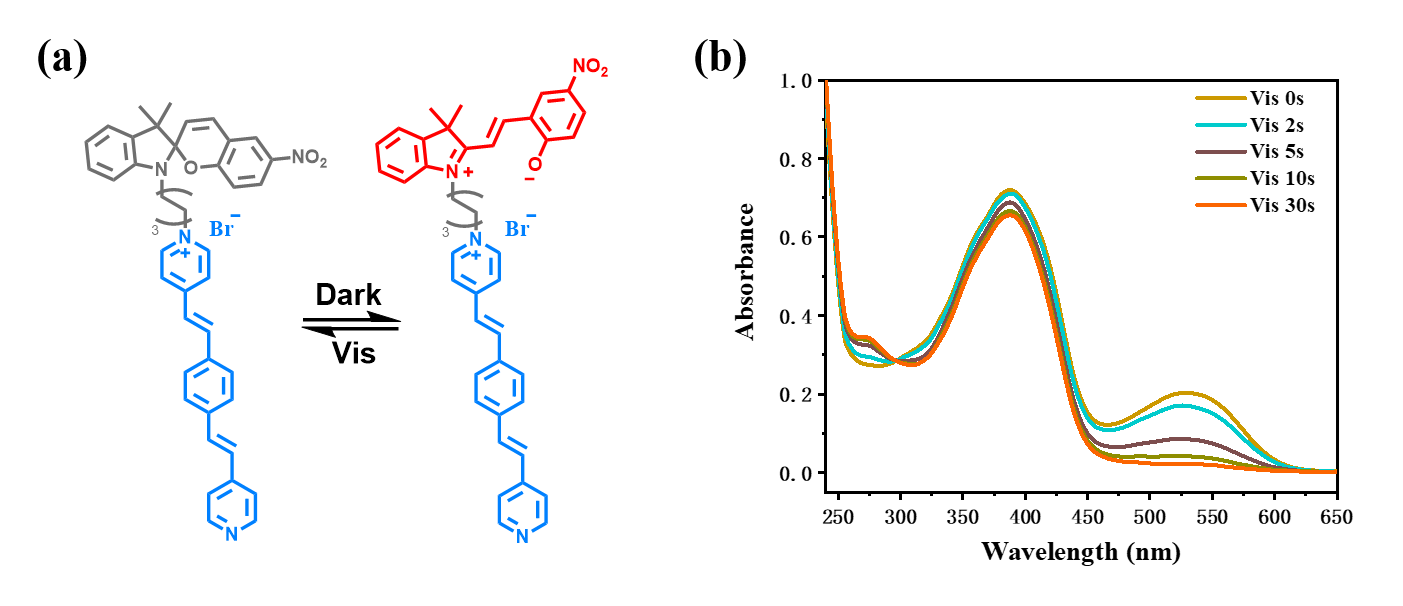
**

Figure S23. (a) The isomerization between ring-closed BPSP and ring-open BPMC under visible light/darkness. (b) UV-Vis absorption spectra of BPMC under visible light ([BPMC] = 2.0×10^-5^ M).

**
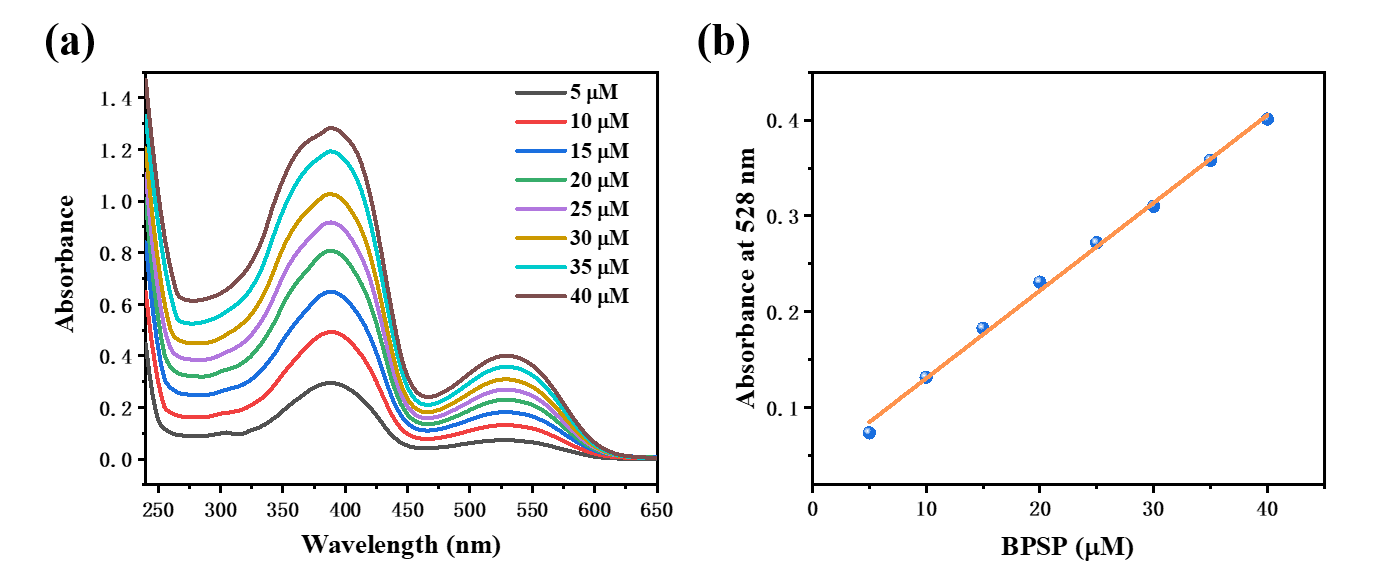
**

Figure S24. (a) UV-Vis absorption spectra of BPMC at different concentrations. (b) The standard curve of BPMC according to the absorption intensity at 528 nm ([BPMC] = 0.5-4.0×10^-5^ M).

**
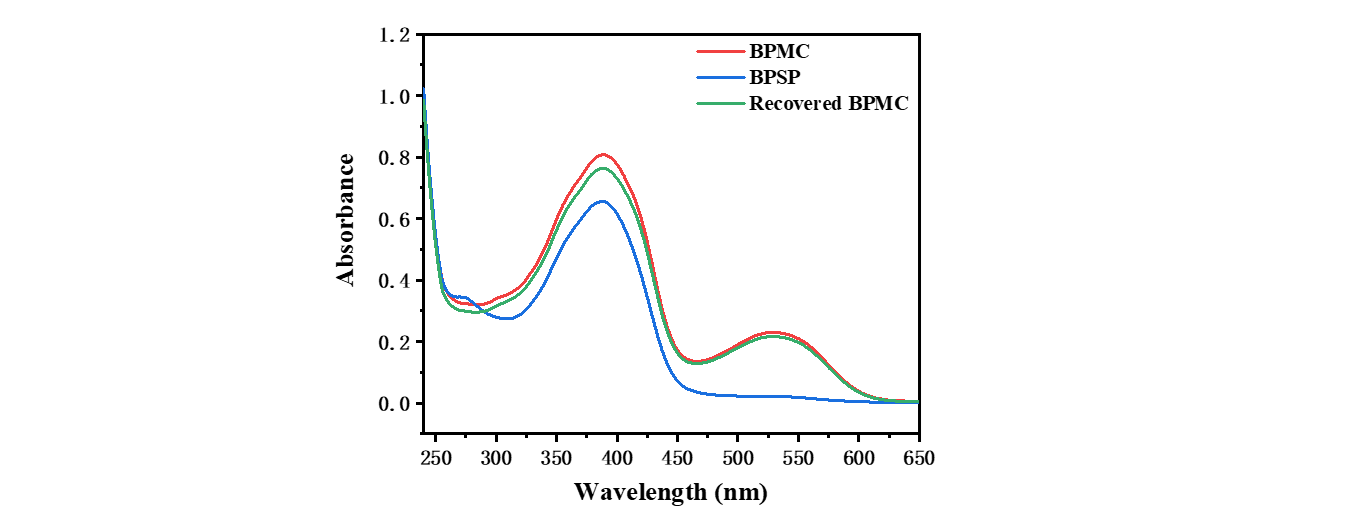
**

Figure S25. UV-Vis absorption spectra of initial BPMC, BPSP after visible light irradiation and recovered BPMC in dark ([BPMC] = 2.0×10^-5^ M, [BPSP] = 2.0×10^-5^ M).

**
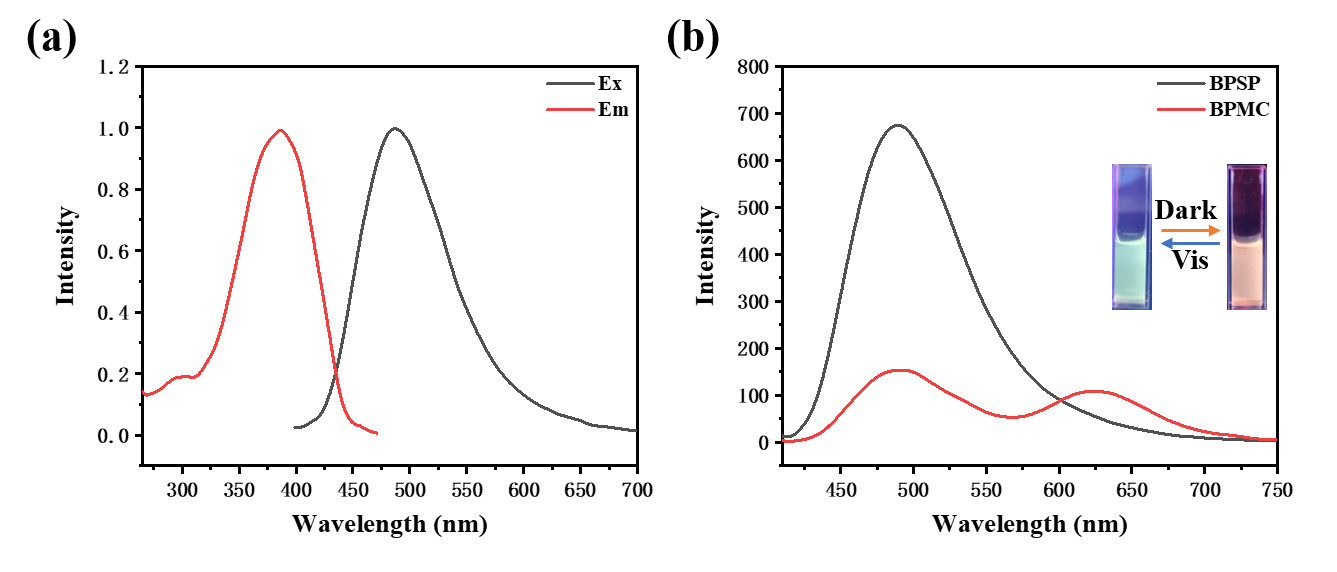
**

Figure S26. (a) The normalized excitation and emission spectra of BPSP. (b) Fluorescence spectra of BPSP and BPMC. Inset: photos of BPSP and BPMC under UV lamp ([BPMC] = 2.0×10^-5^ M, [BPSP] = 2.0×10^-5^ M).

**
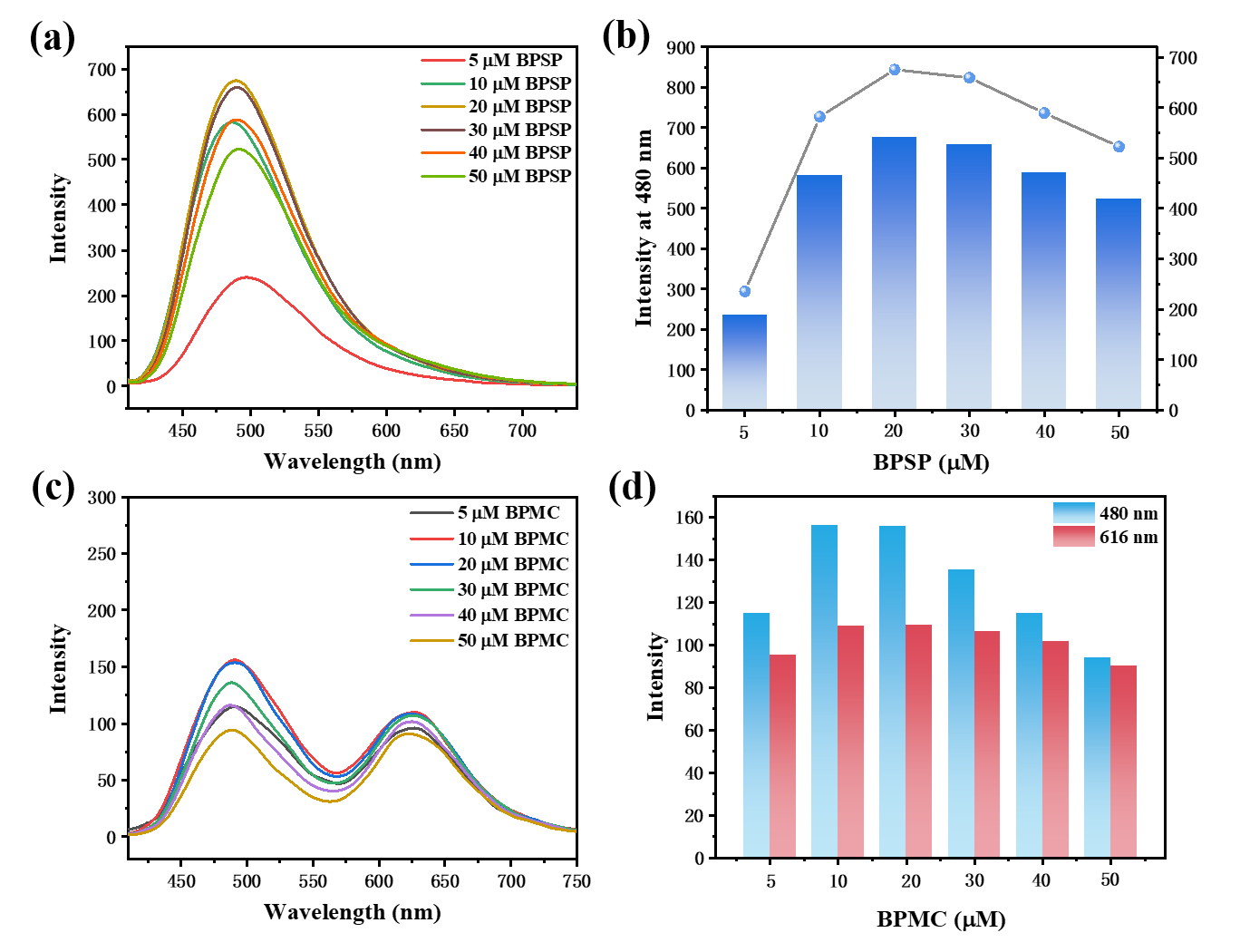
**

Figure S27. (a) Fluorescence spectra of BPSP at different concentrations. (b) Fluorescence intensity of BPSP at 480 nm. (c) Fluorescence spectra of BPMC at different concentrations. (d) Fluorescence intensity of BPMC at 480 nm and 616 nm ([BPSP] = 0.5-5.0×10^-5^ M, [BPMC] = 0.5-5.0×10^-5^ M).

**5. The photo-responsive fluorescence behavior of the BPSP⊂CB[7]**

**
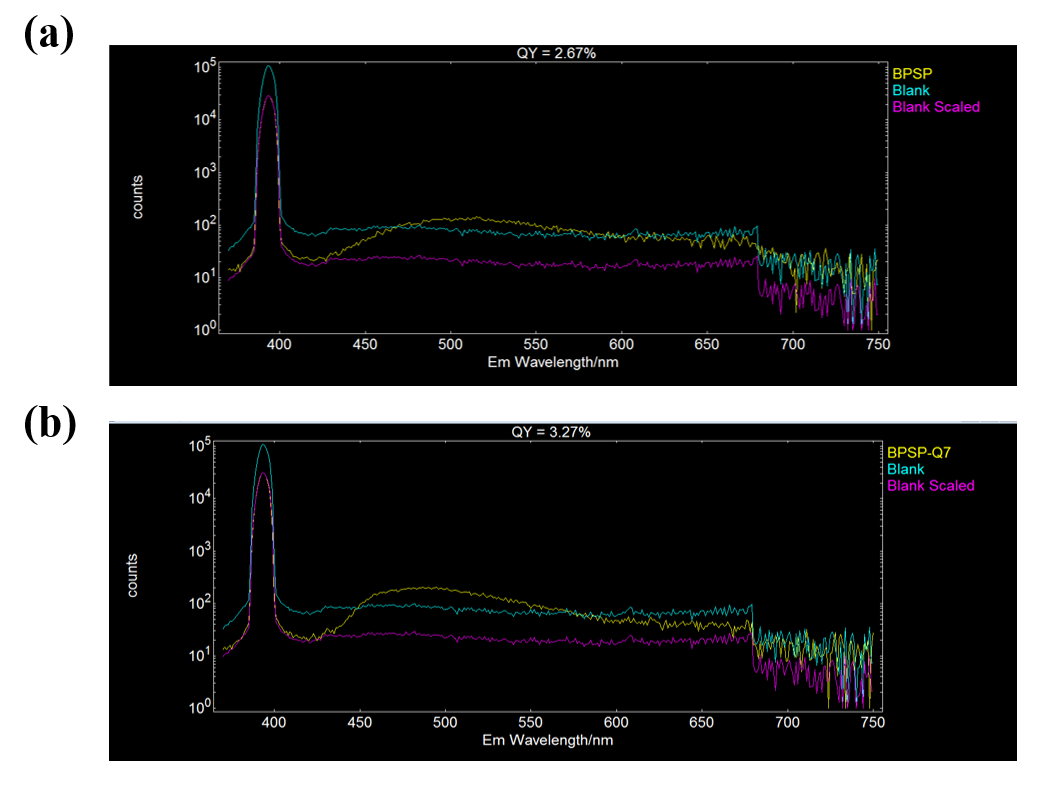
**

Figure S28. The fluorescence quantum yields of (a) BPSP and (b) BPSP⊂CB[7] ([BPSP] = 2.0×10^-6^ M, [CB[7]] = 2.0×10^-6^ M).

**
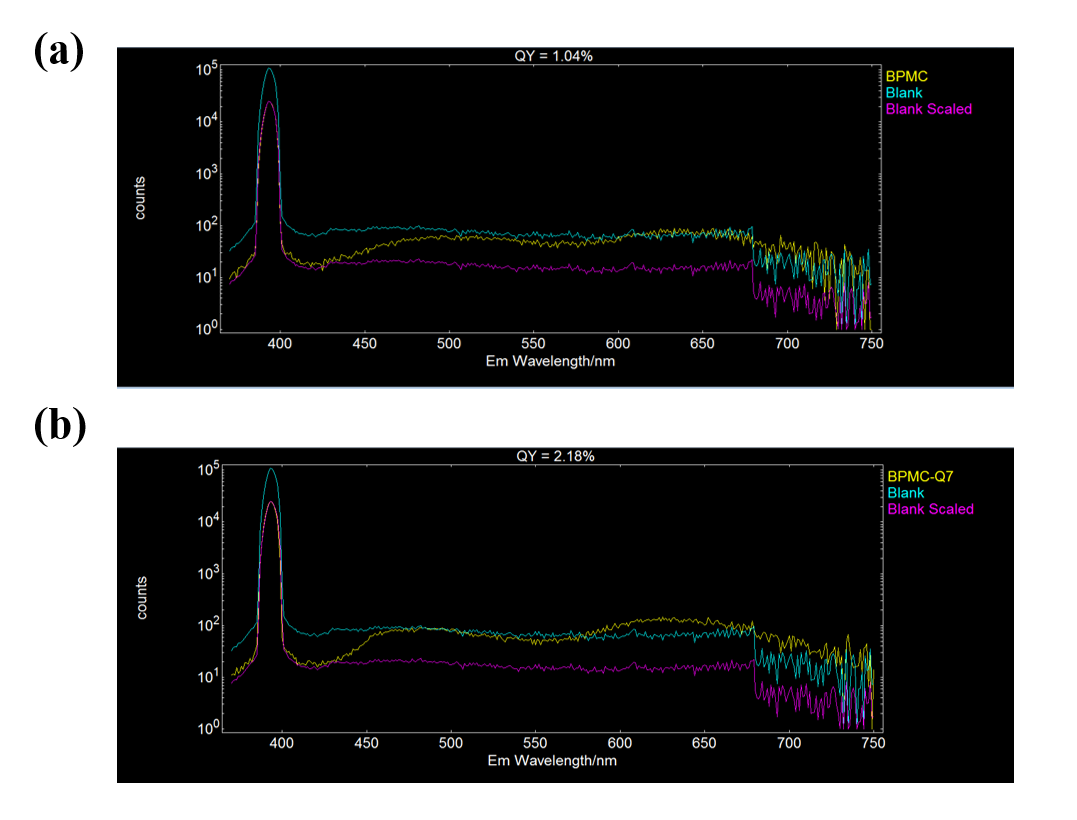
**

Figure S29. The fluorescence quantum yields of (a) BPMC and (b) BPMC⊂CB[7] ([BPMC] = 2.0×10^-6^ M, [CB[7]] = 2.0×10^-6^ M).

**
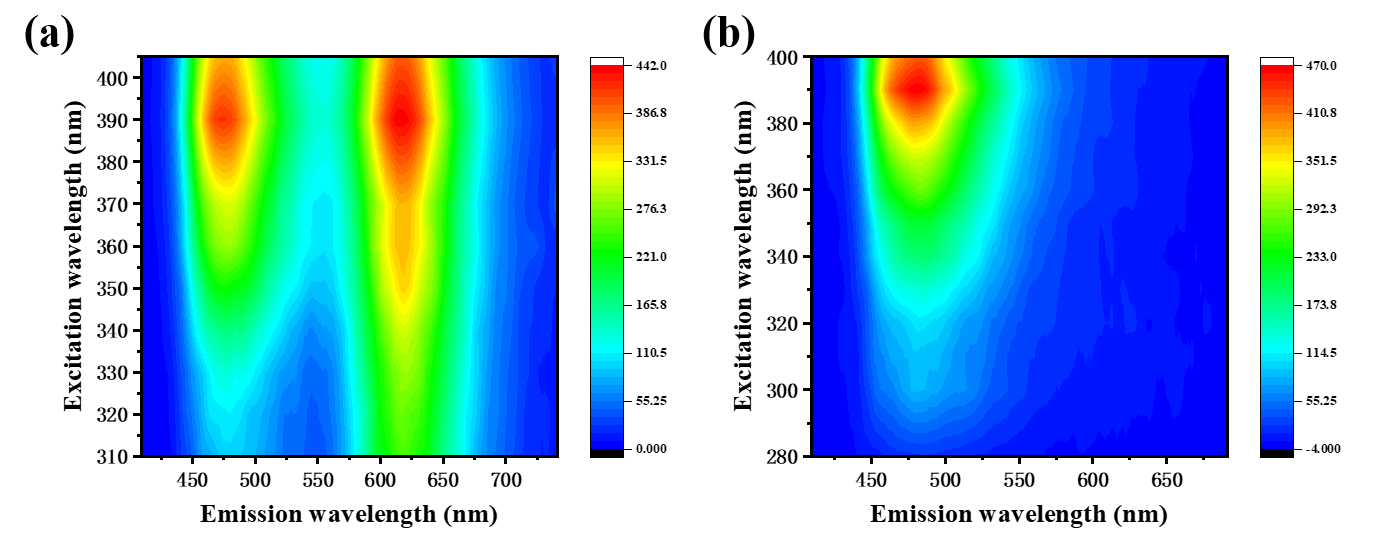
**

Figure S30. Ex-Em mapping spectra of BPMC⊂CB[7] and BPSP⊂CB[7] ([BPMC] = [BPSP] = 2.0×10^-5^ M, [CB[7]] = 2.0×10^-5^ M).


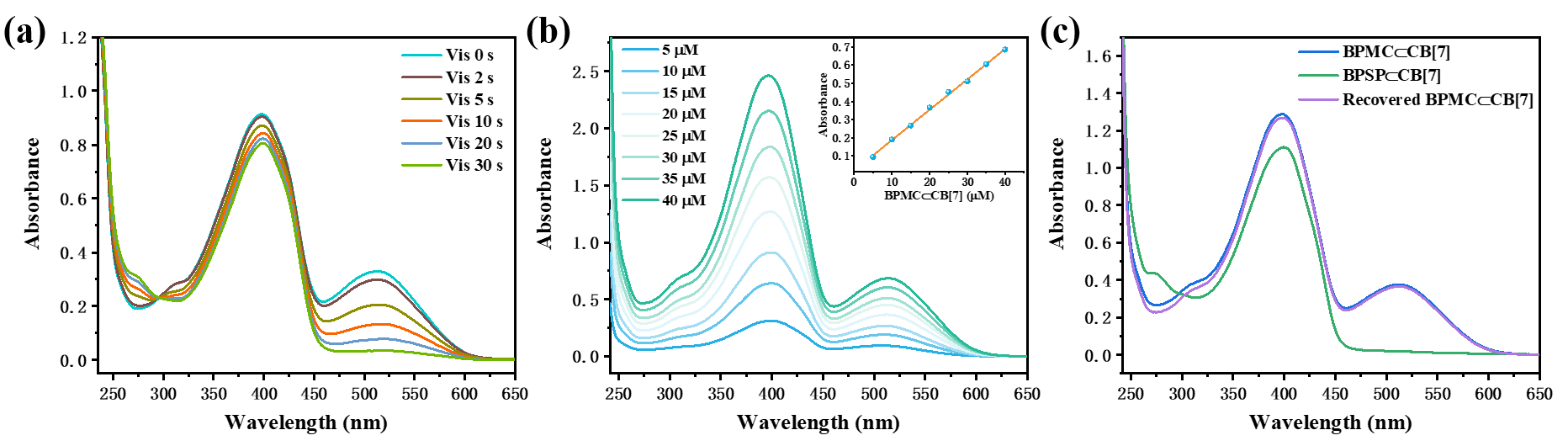


Figure S31. (a) UV-Vis absorption spectra of BPMC⊂CB[7] under visible light ([BPMC] = 2.0×10^-5^ M, [CB[7]] = 2.0×10^-5^ M). (b) UV-Vis absorption spectra of BPMC⊂CB[7] at different concentrations. Insert: The standard curve of BPMC⊂CB[7] ([BPMC] = [CB[7]] = 0.5-4.0×10^-5^ M). (c) UV-Vis absorption spectra of initial BPMC⊂CB[7], BPSP⊂CB[7] after visible light irradiation and recovered BPMC⊂CB[7] in dark ([BPMC] = [BPSP] = 2.0×10^-5^ M, [CB[7]] = 2.0×10^-5^ M).

**6. The photo-responsive fluorescence changes of the BPSP⊂CB[8]**

**
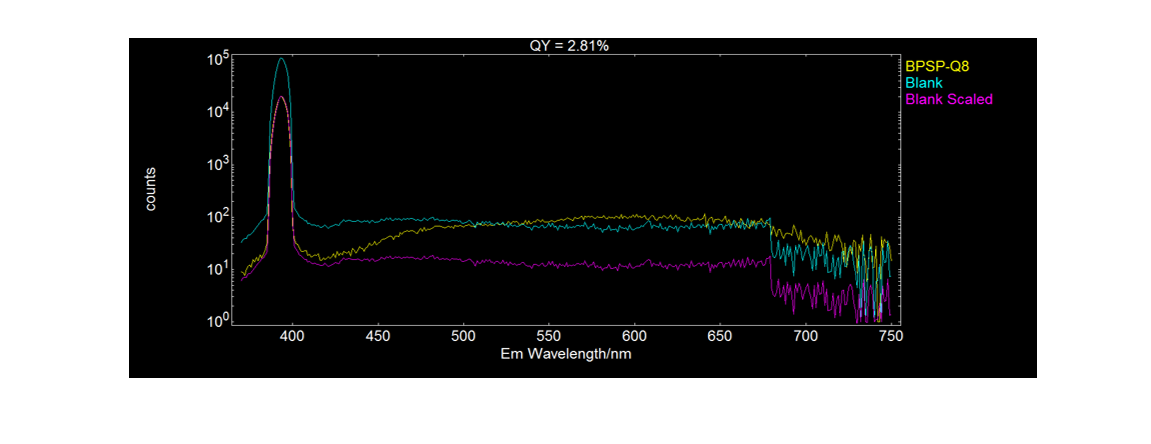
**

Figure S32. The fluorescence quantum yields of BPSP⊂CB[8] ([BPSP] = 2.0×10^-6^ M, [CB[8]] = 2.0×10^-6^ M).

**
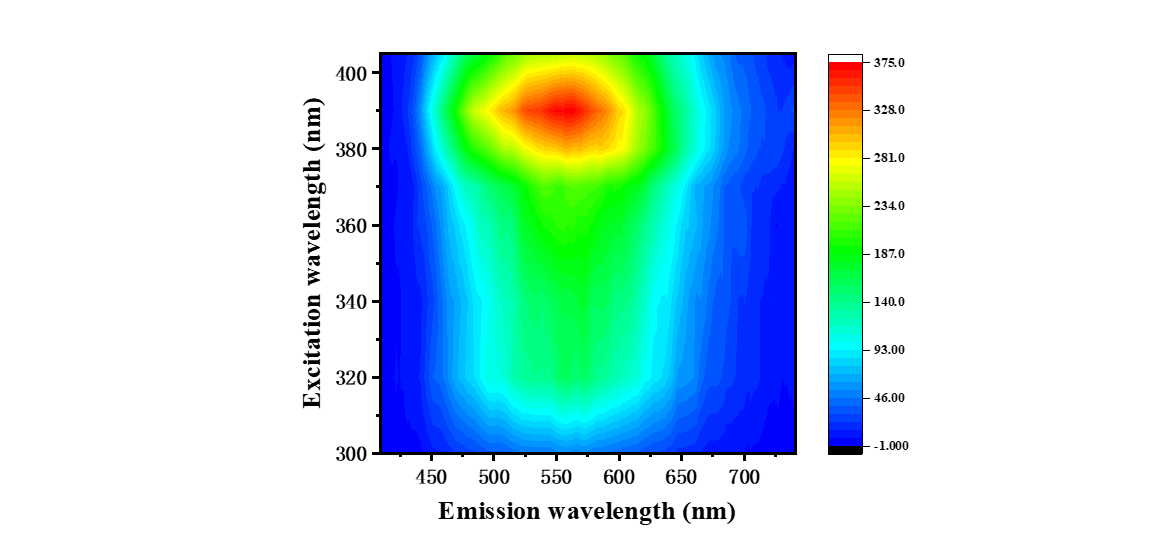
**

Figure S33. Ex-Em mapping spectra of BPSP⊂CB[8] ([BPSP] = 2.0×10^-5^ M, [CB[8]] = 2.0×10^-5^ M).

**
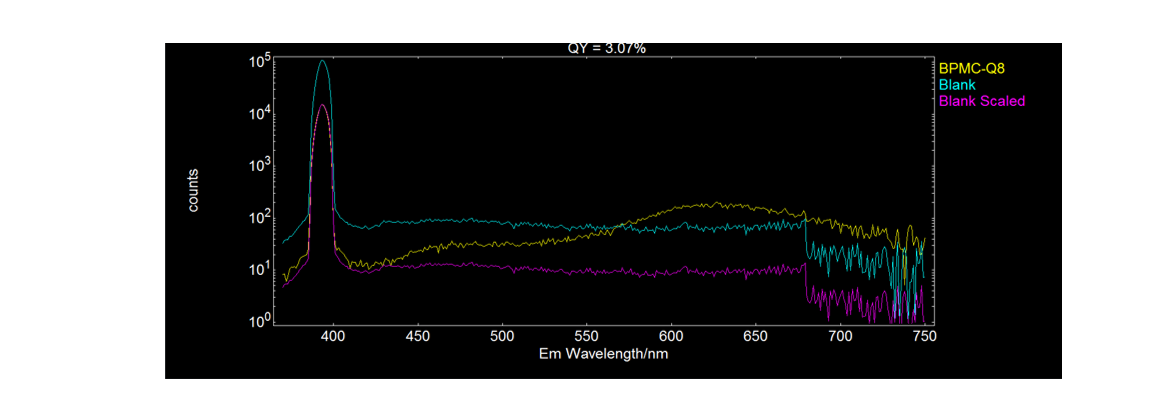
**

Figure S34. The fluorescence quantum yields of BPMC⊂CB[8] ([BPMC] = 2.0×10^-6^ M, [CB[8]] = 2.0×10^-6^ M).

**
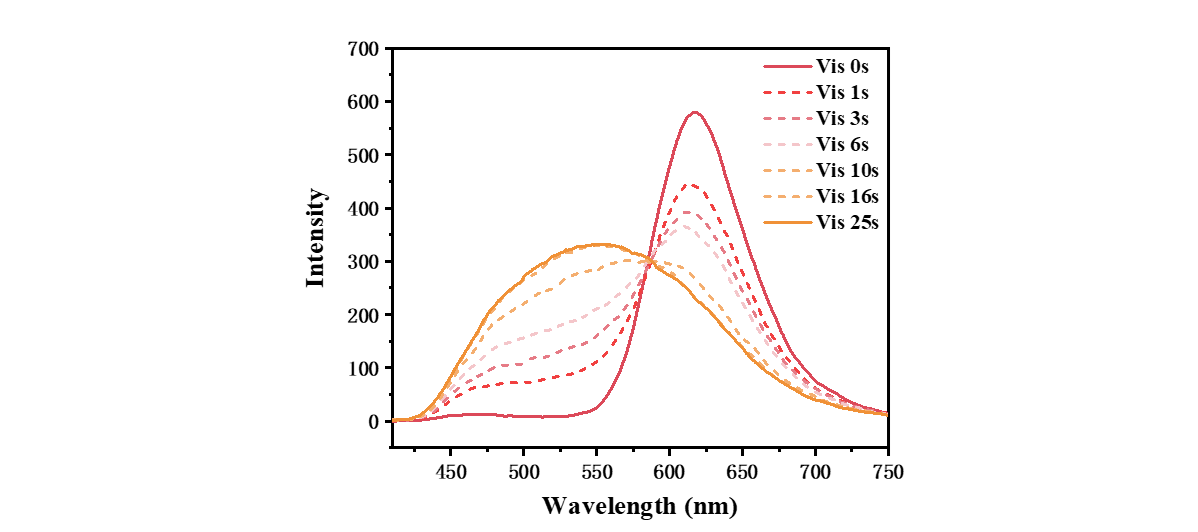
**

Figure S35. Fluorescence spectra of BPMC⊂CB[8] under visible light ([BPMC] = 2.0×10^-5^ M, [CB[8]] = 2.0×10^-5^ M).


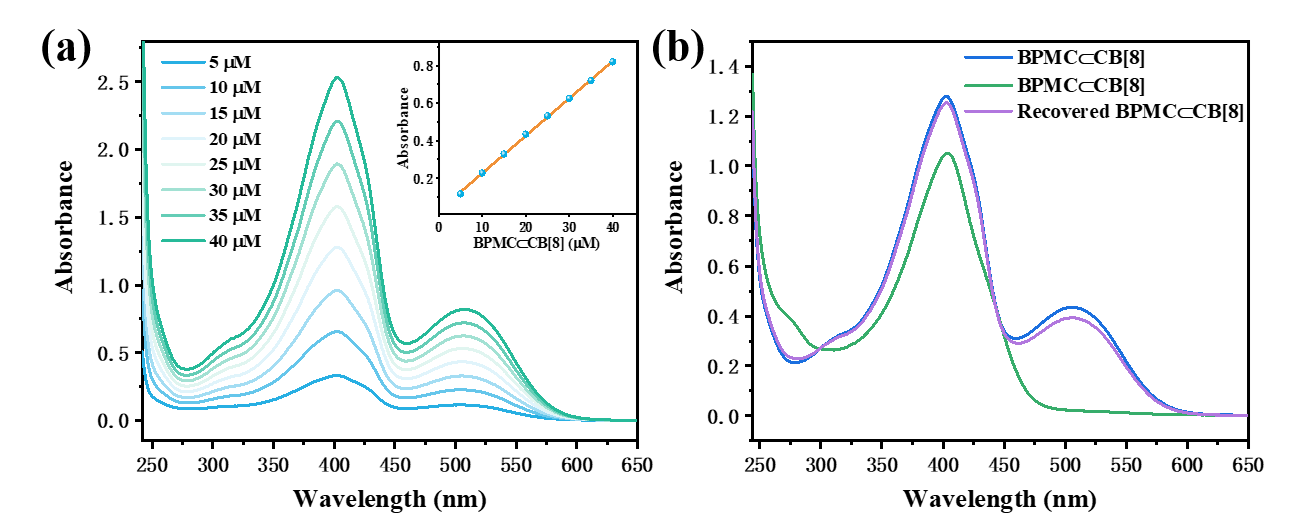


Figure S36. (a) UV-Vis absorption spectra of BPMC⊂CB[8] at different concentrations. Insert: The standard curve of BPMC⊂CB[8] ([BPMC] = [CB[8]] = 0.5-4.0×10^-5^ M). (b) UV-Vis absorption spectra of initial BPMC⊂CB[8], BPSP⊂CB[8] after visible light irradiation and recovered BPMC⊂CB[8] in dark ([BPMC] = [BPSP] = 2.0×10^-5^ M, [CB[8]] = 2.0×10^-5^ M).

**
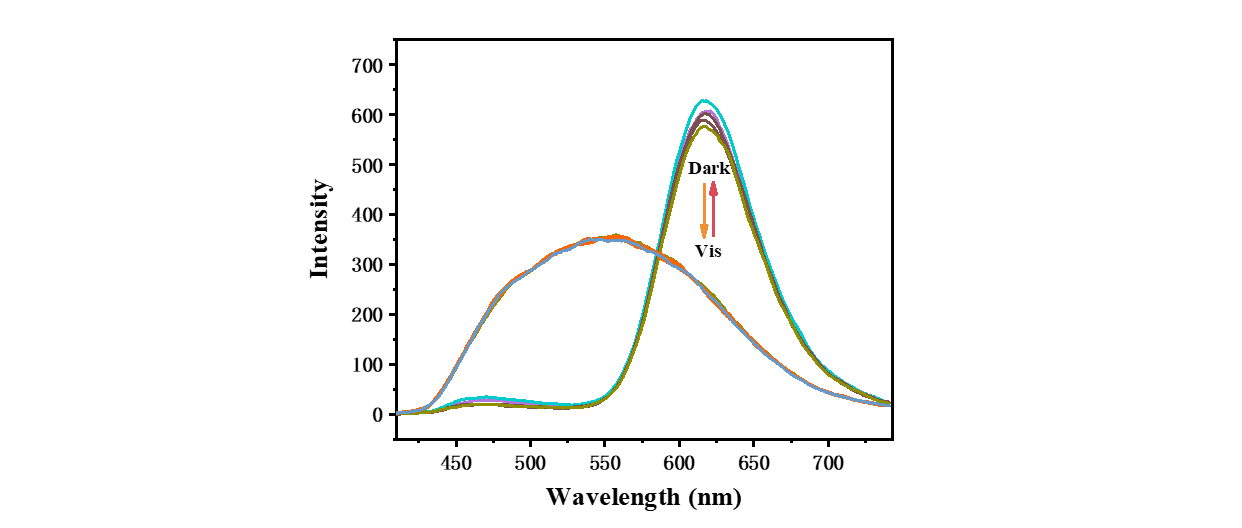
**

Figure S37. Fluorescence spectra of BPSP⊂CB[8] under alternating visible light and darkness ([BPSP] = 2.0×10^-5^ M, [CB[8]] = 2.0×10^-5^ M).

**
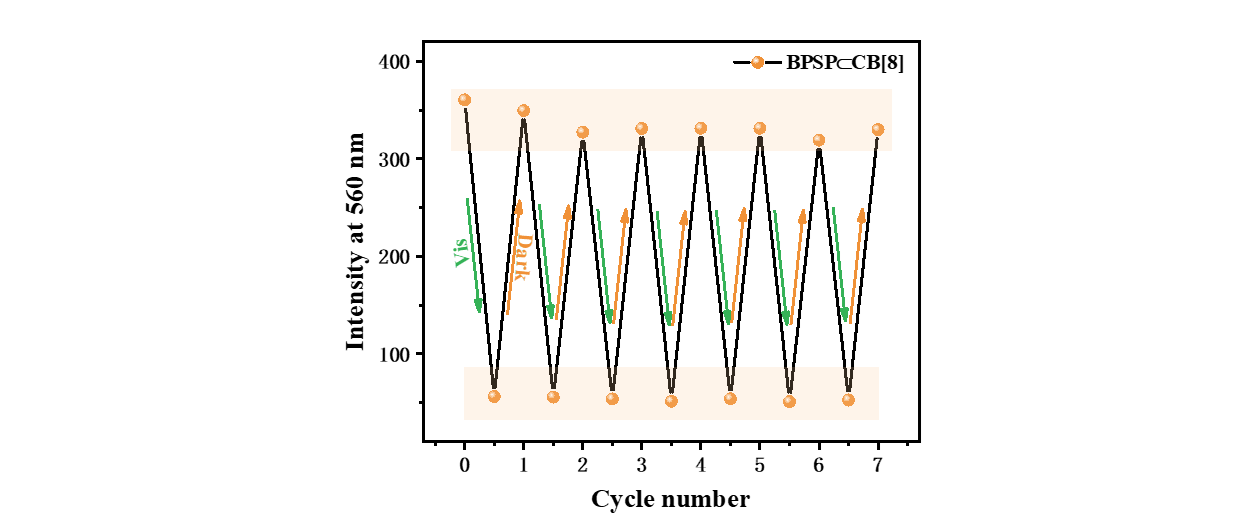
**

Figure S38. The fluorescence intensity changes of BPSP⊂CB[8] at 560 nm under alternating visible light and darkness ([BPSP] = 2.0×10^-5^ M, [CB[8]] = 2.0×10^-5^ M).

**
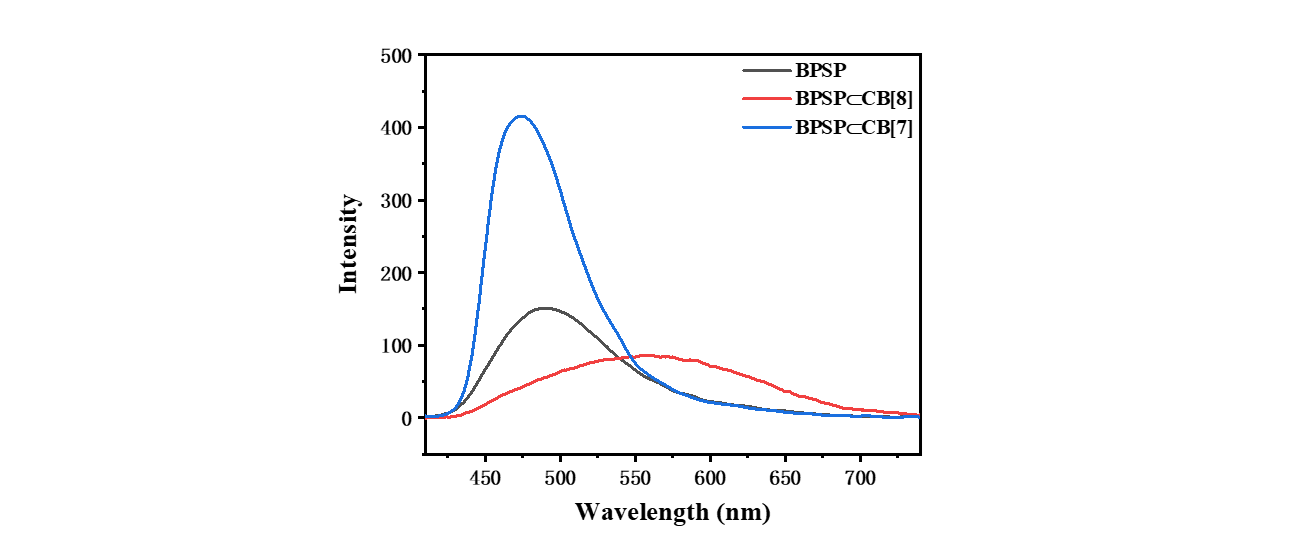
**

Figure S39. Fluorescence spectra of BPSP, BPSP⊂CB[8] and BPSP⊂CB[7] ([BPSP] = 2.0×10^-5^ M, [CB[8]] = 2.0×10^-5^ M, [CB[7]] = 2.0×10^-5^ M).

**7. Reference**

1. Bhowmik, P. K.; Nedeltchev, A. K.; Han, H., Synthesis, optical, and thermal properties of conjugated, bispyridyl and tetrapyridyl compounds by Knoevenagel reaction. *Tetrahedron Letters* **2007,** *48* (31), 5383-5387.

2. Kim, J.; Lee, Y. J.; Ku, K. H.; Kim, B. J., Effect of Molecular Structure of Photoswitchable Surfactant on Light-Responsive Shape Transition of Block Copolymer Particles. *Macromolecules* **2022,** *55* (18), 8355-8364.
